# Hypoxic-preconditioned mesenchymal stem cell-derived small extracellular vesicles inhibit neuronal death after spinal cord injury by regulating the SIRT1/Nrf2/HO-1 pathway

**DOI:** 10.3389/fphar.2024.1419390

**Published:** 2024-08-23

**Authors:** Jian Rao, Haishu Xie, Zeyan Liang, Zhelun Yang, Pingping Chen, Maochao Zhou, Xiongjie Xu, Yike Lin, Fabin Lin, Rui Wang, Chunhua Wang, Chunmei Chen

**Affiliations:** ^1^ Department of Neurosurgery, Fujian Medical University Union Hospital, Fuzhou, Fujian, China; ^2^ Fujian Neurosurgical Institute, Fuzhou, Fujian, China

**Keywords:** spinal cord injury, mesenchymal stem cells, small extracellular vesicles, silent information regulator 1, oxidative stress

## Abstract

**Background:**

Oxidative stress and apoptosis of neurons significantly contribute to the pathophysiological cascade of spinal cord injury (SCI). However, the role of hypoxic-preconditioned mesenchymal stem cell-derived small extracellular vesicles (H-sEVs) in promoting SCI repair remains unclear. Hence, the present study aims to investigate the regulatory effects of H-sEVs on neuronal oxidative stress and apoptotic responses following SCI.

**Methods:**

The administration of H-sEVs of SCI rats was assessed using behavioral evaluations such as Basso-Beattie-Bresnahan (BBB) scores, neuroelectrophysiological monitoring, and Catwalk gait analysis. Indices of oxidative stress (including superoxide dismutase [SOD], total antioxidant capacity [T-AOC], and malondialdehyde [MDA]) were measured. Neuronal survival was evaluated through Nissl staining, while the expression level of sirtuin 1 (SIRT1) was examined using immunohistochemical staining. Additionally, histological evaluation of lesion size was performed using hematoxylin-eosin (HE) staining. Tunel cell apoptosis staining and analysis of apoptosis-associated proteins (B-cell lymphoma-2 [Bcl2] and BCL2-Associated X [Bax]) were conducted through immunofluorescence staining and western blot, respectively. Furthermore, the model of oxidative stress was established using PC12 cells, and apoptosis levels were assessed via flow cytometry and western blot analysis. Importantly, to ascertain the critical role of SIRT1, we performed SIRT1 knockout experiments in PC12 cells using lentivirus transfection, followed by western blot.

**Results:**

Using those behavioral evaluations, we observed significant functional improvement after H-sEVs treatment. Nissl staining revealed that H-sEVs treatment promoted neuronal survival. Moreover, we found that H-sEVs effectively reduced oxidative stress levels after SCI. HE staining demonstrated that H-sEVs could reduce lesion area. Immunohistochemical analysis revealed that H-sEVs enhanced SIRT1 expression. Furthermore, Tunel cell apoptosis staining and western blot analysis of apoptosis-related proteins confirmed the anti-apoptotic effects of H-sEVs. The PC12 cells were used to further substantiate the neuroprotective properties of H-sEVs by significantly inhibiting neuronal death and attenuating oxidative stress. Remarkably, SIRT1 knockout in PC12 cells reversed the antioxidant stress effects induced by H-sEVs treatment. Additionally, we elucidated the involvement of the downstream Nrf2/HO-1 signaling pathway.

**Conclusion:**

Our study provides valuable insights into the effects of H-sEVs on neuronal oxidative stress and apoptosis after SCI. These findings underscore the potential clinical significance of H-sEVs-based therapies for SCI.

## Introduction

The central nervous system (CNS), which consists of the brain and spinal cord, is the most vulnerable and sensitive system in the body. Spinal cord injury (SCI) is a catastrophic injury to the CNS that causes sensory and motor dysfunction and even affects other tissues or organs (McDonald and Sadowsky, 2002). The occurrence of SCI can lead to impaired nerve function below the site of injury, resulting in a neurogenic bladder and subsequent urine retention (de Groat and Yoshimura, 2012). Additionally, it may also give rise to intestinal obstruction (White et al., 2020). Due to its high disability rate and fatality rate, it brings a heavy economic burden to the family and society (Ahuja et al., 2017b). According to statistics, by 2020, the average annual increase of SCI patients in the world is about 600,000–940,000, with the total incidence as high as 10.5/100,000 (Rahimi-Movaghar et al., 2013; Kumar et al., 2018). Although the incidence is different in different countries and regions, the pathogenic factors are different, mainly including car accidents, falls, and tumor compression (Rahimi-Movaghar et al., 2013; Jain et al., 2015). SCI is divided into primary and secondary injuries. Under the direct action of external violence, the primary injury of the spinal cord will result in axon rupture and blood vessel rupture and bleeding at the injured site (Ahuja et al., 2017a). After primary injury, glial cells and T cells are activated and inflammatory cytokines such as tumor necrosis factor-α (TNF-α) and interleukin-1-β (IL-1β) are released due to local inflammation, hematoma, and ischemia (Anderson et al., 2016).

The surge of reactive oxygen species (ROS) and nitric oxide (NO) alters the damaged microenvironment, leading to oxidative stress, apoptosis, and even necrosis of endothelial cells and neurons (Venkatesh et al., 2019; Anjum et al., 2020). Oxidative stress plays an important role in the pathological process of SCI. Studies have shown that the levels of total antioxidant capacity (T-AOC), glutathione (GSH), malondialdehyde (MDA), superoxide dismutase (SOD), and catalase after SCI are abnormal, resulting in severe oxidative damage (Jia et al., 2012; Fatima et al., 2015). Oxidative stress increases ROS and NO levels, and high levels of ROS and NO lead to cytotoxic effects, vasodilation, neuronal apoptosis, breakdown of the blood-spinal barrier, neurological dysfunction, and neurodegeneration (Matsuyama et al., 2014). Oxidative stress, as the fuse of inflammation and apoptosis pathway, triggers the cascade reaction after SCI, further aggravating the microenvironment after injury (Hall, 2011). Therefore, inhibition of oxidative stress can significantly improve the microenvironment after SCI and promote spinal cord repair. It is considered to be one of the most important strategies to prevent or improve the progress of SCI.

At present, the clinical treatment of SCI includes surgery, drug therapy, biomaterials, and stem cell transplantation (Rowland et al., 2008). Otherwise, the therapeutic effect is not satisfactory. Studies have shown that therapeutic properties of mesenchymal stem cells (MSCs) may depend on paracrine action of cells (Phan et al., 2018; Nakazaki et al., 2021). MSCs are considered the most promising treatment because the transplanted cells can provide nutritional support, regulate inflammation, and promote nerve regeneration (Si et al., 2011). However, there are also some limitations, such as low cell survival rate after transplantation, strong immune rejection, and tumorigenicity (Flowers and Martin, 2015). Extracellular vesicles (EVs) are secreted actively by cells, and studies have shown that stem cells secrete the largest amount of EVs (Kourembanas, 2015; van Niel et al., 2018). Due to its low immunogenicity and tumorigenicity, EVs have become a promising therapeutic approach, overcoming the limitations of MSCs (Kourembanas, 2015). EVs are derived from cells, serum, and other body fluids and are involved in signaling between cells (Kalluri and LeBleu, 2020). Small EVs are a group of lipid bilayer cup vesicles with a diameter of 30–200 nm, which contain a variety of bioactive substances, including RNA, DNA, proteins, lipids, and cell metabolites (Jeppesen et al., 2019). Under physiological and pathological conditions, they can carry goods between cells and have a critical impact on communication between cells. Peng et al. (Peng et al., 2021) demonstrated that microglia-derived exosomes inhibit oxidative stress and protect endothelial cells in SCI. The damage microenvironment in tissues is often in a hypoxic state, so the EVs secreted by stem cells under hypoxic preconditioning can better simulate the damage microenvironment *in vivo* (Bae et al., 2018). It has shown that small extracellular vesicles derived from hypoxic-preconditioned mesenchymal stem cells (H-sEVs) can help repair myocardial cell damage (Zhu et al., 2018). However, the mechanism of efficacy of H-sEVs in oxidative stress of SCI remains unclear.

Silent information regulator 1 (SIRT1) is a Class III histone deacetylase that is a nicotinamide adenine dinucleotide-dependent enzyme. In mammals, this family consists of seven members (SIRT1-7), and the function of SIRT1 has been extensively studied (Wu et al., 2022). Many studies have demonstrated that SIRT1 can widely regulate inflammatory response, oxidative stress DNA repair, apoptosis, and energy metabolism in a variety of cells (Jiao and Gong, 2020; Liu et al., 2021). Previous studies have shown that SIRT1 regulates oxidative stress and inflammation through a variety of signaling pathways and alleviates oxidative stress and inflammation in a variety of diseases, including renal, cardiovascular, and cerebrovascular ischemia-reperfusion injury (Wu et al., 2018; Mao et al., 2022; Mei et al., 2022). Numerous studies have shown that SIRT1 can relieve oxidative stress and inflammation by regulating Nuclear factor-kappa B (NF-kB), Adenosine 5‘-monophosphate (AMP)-activated protein kinase (AMPK), and Forkhead box O1 (FOXO1) (Gao et al., 2020; Feng et al., 2022; Song et al., 2022). Moreover, it can protect neuronal damage and apoptosis (Cui et al., 2021). Specifically, SIRT1 plays a vital role in alleviating secondary damage to the central nervous system and regulates downstream transcription factors, including Nuclear related factor 2 (Nrf2) and NF-kB, to reduce oxidative stress and apoptosis (Singh and Ubaid, 2020). Nrf2 is a major transcription factor that regulates antioxidant response and has become a potential therapeutic target for inflammatory diseases. Dysregulation of Nrf2 leads to decreased antioxidant enzymes and detoxification enzymes, which are associated with increased secondary injury in SCI (Guo et al., 2022). Heme oxygenase-1 (HO-1) is a key downstream stress-inducing protein of Nrf2, which also plays an anti-oxidative stress and apoptosis (Zhao et al., 2019). Therefore, we speculate that SIRT1 may play a key role in the improvement of oxidative stress after SCI by H-sEVs, and the downstream proteins Nrf2 and HO-1 regulated by SIRT1 may also be involved.

The present study systematically examined the impact of H-sEVs on functional recovery following SCI by ameliorating oxidative stress and inhibiting apoptosis. The neuroprotective effect of H-sEVs was further demonstrated in PC12 cells. Additionally, SIRT1 has been identified as a crucial protein in mitigating oxidative stress post-SCI, as the ablation of SIRT1 can reverse the anti-oxidative stress effect of H-sEVs. These findings enhance our understanding of the mechanisms underlying the use of H-sEVs for promoting SCI repair and offer new insights and theoretical foundations for the translation and application of H-sEVs in clinical treatment for SCI.

## Materials and methods

### Animal preparation and experimental grouping

Adult male SD rats (weight: 200–250 g, age: 6–8 weeks, n = 60) were provided by SPF Biotechnology Co., Ltd. (Beijing, China). All the rats were kept in a suitable environment (temperature 22°C–24°C, humidity 60%–80%) and had free access to food and water in the cages. The keepers were completely unaware of the experimental protocol. All experimental protocols were approved by the Fujian Medical University Institutional Animal Care and Use Committee (Ethical approval number: FJMU IACUC 2021-0501). The rats were randomly divided into Sham group, SCI group, and SCI + H-sEVs group with 20 rats in each group. The rats in the SCI + H-sEVs group were injected with 500 μL (0.2 μg/μL) of H-sEVs each time, and the rats in the SCI group were injected with equal volume of PBS. H-sEVs were first injected through the caudal vein 2 h after SCI and then every 24 h thereafter for a total of three injections.

### Isolation, culture, and identification of MSCs

Bone marrow was extracted from femur and tibia of young male SD rats (weight: 80–100 g, age: 2–3 weeks, n = 5) for isolation and culture of primary MSCs. The lower femur and tibia were isolated under sterile conditions, the bone marrow was carefully flushed out using complete medium (high-glucose DMEM, 10% FBS, and 1% penicillin/streptomycin double antibody), and the suspension was gently blown away. After centrifugation of 300 g for 5 min, the supernatant was discard. After re-suspension precipitation using complete medium, the cells were inoculated in cell culture flasks and cultured in an incubator at 37°C and 5% CO_2_. The medium was first changed 24 h after inoculation, washed with PBS to remove unadherent cells, and then changed every 3 days. After 10 days, a microscopically well developed fibroblast-like cell colony was visible. The cells were trypsinized and counted (adjusted cell density 1×10^5^ cells/mL), then passed into a new culture flask for further amplification. The medium was changed every 2–3 days until the cell fusion rate was 70%–80%, and the next experiment was carried out.

MSCs surface markers were identified by flow cytometry: P4-7 generations of MSCs at logarithmic growth stage were taken, trypsinized, washed with PBS for 2–3 times, and the cell concentration was adjusted to 1×10^6^ cells/mL using PBS. MSCs were then incubated with the anti-CD29 (Cat# 12-0291-82, ThermoFisher Scientific), anti-CD90 (Cat# 17–0900-82, ThermoFisher Scientific), anti-CD34 (Cat# 11–0341-82, ThermoFisher Scientific), and anti-CD45 (Cat# 561587, BD Bioscience) at 37°C for 30 min, away from light. Then, wash with PBS for 2–3 times, 300 g, centrifuge for 5 min. Finally, sample analysis was performed using BD FACS Celesta cytometry (BD, USA). All data analysis was performed using FlowJo V10 (Stanford University, Palo Alto, CA, USA).

Differentiation potential identification of MSCs: MSCs of P4-7 generations were trypsinized and inoculated into 6-well plates. Osteogenic differentiation, lipogenic differentiation, and chondrogenic differentiation were induced and identified according to the MSCs induction differentiation kit (Cyagen, China) according to the protocol provided by the reagent supplier. Osteogenic differentiation, lipogenic differentiation, and chondrogenic differentiation were stained with alizarin red, oil red O, and alcian blue, respectively, and washed with PBS for 2–3 times to wash away the dye solution. The induced differentiation of MSCs was observed under a light microscope (Leica, Germany).

### Isolation and identification of H-sEVs

When MSCs of P4-P7 generation reached 70% confluence, and the original medium was removed, washed with PBS for 2–3 times to remove the residual medium, and replaced with FBS complete medium containing 10% exosomes free (Cyagen, China). MSCs were cultured in a hypoxic workstation (DWS, UK) at 37°C, 1% O_2_, 5% CO_2_, and 94% N_2_ for 48 h. Cell supernatant was collected and centrifuged at 4°C for 10 min at 300 g to remove dead cells. Supernatant was collected, 2000 g and centrifuged at 4 °C for 10 min to remove cell debris. Supernatant was collected at 10,000 g and centrifuged at 4°C for 30 min to remove organelles and large diameter vesicles. The supernatant was collected and slowly added to the ultracentrifuge tube containing 4 mL 30% sucrose layer. The ultracentrifuge tube was centrifuged at 110,000 g at 4°C for 70 min using an ultracentrifuge (Beckman, USA). After two repetitions, the H-sEVs were resuspended with PBS and finally filtered by a 0.22 μm filter (Merck, USA) and stored in sterile EP tubes for subsequent experiments.

Western Blot (WB) method for identification of markers of H-sEVs: The extracted H-sEVs and exosome cracking solution were mixed in a 1:1 ratio according to the volume ratio, and then quickly and repeatedly blown on ice for 1 min and left for 10 min. After centrifugation of 12,000 g at 4 °C for 10 min, the precipitation was discard, and the supernatant was taken. The protein concentration was determined by BCA kit (Beyotime, China). Then the supernatant was added a loading buffer with a volume ratio of 1:4 to adjust the concentration uniformly. The samples with the same concentration were heated in boiling water at 100 °C for 5 min to denaturate, and then 20 μg protein and marker were added with prefabricated glue (ebio-ace, China). Then electrophoresis was performed at constant pressure 160 V for 40 min. After electrophoresis, the proteins were transferred to the NC membrane using a sandwich method at 110 V wet for 1 h (the time was adjusted according to the molecular weight of the specific target protein). They were incubated in a shaker at room temperature for 2 h using a closed solution (5% skim milk powder). Primary antibodies anti-CD9 (Cat# SAB4503606, Merck), anti-CD63 (Cat# PA5-92370, ThermoFisher Scientific), and anti-TSG101 (Cat# MA1-23296, ThermoFisher Scientific) were then added and incubated at 4 °C overnight. On the second day, primary antibody was discarded, and the membrane was washed with TBST for 3 times, 5 min each time. After incubation with the goat anti-rabbit secondary antibody (HRP) or goat anti-mouse secondary antibody (HRP) at room temperature for 1 h, the membrane was washed with TBST for 3 times, 10 min each time. Finally, using the enhanced ECL kit (Beyotime, China), the developing droplets were prepared and added to the front of the NC membrane. After reacting for several seconds, the droplets were exposed under the e-Blot gel imager (e-BLOT, China).

Nanoparticle tracking analysis (NTA) by H-sEVs: In order to analyze the size and concentration distribution of extracellular vesicles, PBS was used to dilute H-sEVs 1000 times, and NTA (NanoFCM, China) was used for analysis and detection. Three samples were measured for each sample. Data analysis was performed using NanoSight NTA 3.4 software (NanoSight, UK), and the concentration and size distribution of H-sEVs were drawn according to the detection results.

Transmission electron microscopy (TEM) by H-sEVs: The Formvar membrane was absorbed for 20 min by using 10 μL H-sEVs suspension. During the cleaning process, the Formvar membrane was kept facing down and the Formvar membrane was kept moist while the other side was dry. The copper mesh was placed on the 5 μL 1% glutaraldehyde liquid drop for 5 min. After the cleaning with double steaming water, 50 μL of the dyeing solution was added to the dioxalate uranium solution for 5 min, and then methyl cellulose was added. The excess liquid was gently sucked on the filter paper and dried in the air for 5–10 min. The dried copper mesh was placed in the box and the shape and size of H-sEVs were observed by TEM (Hitachi, Japan) at 100 KeV.

### Establishment and grouping of SCI rat models

Adult male SD rats (weight: 200–250 g, age: 6–8 weeks, n = 60) were abstinent from food and water 24 h before surgery. Inhalation anesthesia was performed with isoflurane (induction concentration 3%–4%, maintenance concentration 1.5%–2%). After the rats were completely anesthetized and no cardiac arrest was confirmed, the rats were fixed on the test bench in prone position, and iodophor was used for disinfection of the skin. A longitudinal incision of about 4 cm was made at the highest point of the posterior median line of the rat’s back kyphosis, and the tissue was separated layer by layer in a sterile environment. The highest point of the kyphosis corresponded to the T10 lamina. The T10 lamina was removed and the spinal cord was exposed (Supplementary Figure S1A: yellow box mark). T10 is the median spinous process. The spinal cord impactor device (RWD, China) was used to strike the spinal cord at an instantaneous velocity of 2 mm in diameter and 0.5 m/s for 1 s. At the moment of the blow, a significant hematoma was observed by the naked eye (Supplementary Figure S1C: marked in yellow frame). After a few seconds of implantation, spasmodic contraction of hind limbs and spasmodic rotation of tail were observed (Supplementary Figure S1B), followed by decreased muscle tension of both hind limbs and tail. BBB score after waking was 0, indicating successful modeling. After operation, the incisions of rats were disinfected with iodophor, the tissue was closed layer by layer, and the rats were fed in a single cage. Intramuscular injection of 80,000 U penicillin was given once a day for 3 days after surgery to prevent infection. Bladder massage was used to urinate twice a day until the bladder function recovered.

Forty-two rats were randomly divided into three groups with 14 rats in each group. They were divided into three groups. Sham group: Only T10 lamina was resected without any treatment. SCI group: After SCI, rats were injected with an equal volume of PBS intravenously. SCI + H-sEVs group: After SCI, H-sEVs with protein quantity of 100 μg was injected into caudal vein 2 h after surgery for three consecutive days. Subsequently, the rats were euthanized on the seventh day after surgery, and spinal cord tissue samples and protein samples were obtained for follow-up experiments.

Eighteen rats were selected blind and randomly divided into three groups with six rats in each group. They were divided into three groups. Sham group: Only T10 lamina was resected without any treatment. SCI group: After SCI, rats were injected with an equal volume of PBS intravenously. SCI + H-sEVs group: After SCI, H-sEVs with protein quantity of 100 μg was injected into caudal vein 2 h after surgery for three consecutive days. This part was used to study the recovery of hind limb motor function in rats.

### SCI scores of hind limb motor function in rats

The Basso, Beattie and Bresnahan Locomotor Rating Scale (BBB Scale) is an effective tool for evaluating hind limb motor function in rats to reflect spinal cord nerve function (Basso et al., 1995). The motor function of hind limbs was evaluated at 1, 3, 7, 14, 21, and 28 days after surgery. Each rat was placed in the same open field for free movement and observed for 5 min. The walking of the hips, knees, ankles, toes, trunk balance and coordination, and tail coordination were observed. Double-blind method was used for each measurement, that is, two researchers who were proficient in the scoring criteria and unaware of the experimental scheme observed and scored independently.

### Electrophysiological monitoring

The motor evoked potentials (MEPs) at the 28th day after SCI was evaluated by electrophysiological monitoring apparatus (Medtronic, USA) in order to assess the overall neurological function of rats at the same time (Sheng et al., 2021). Rats were anesthetized by inhalation using isoflurane (maintenance concentration 1.5%–2%). After anesthesia, the stimulation electrode placed under the skull were used to stimulate motor areas of the cerebral cortex. The recording electrode was placed in the flexor femoris biceps muscle. The reference electrode was placed in the tendon at the distal end of the hindlimb muscle. And the grounding electrode was placed subcutaneously. One-power wave stimulation of 0.5 mA, 0.5 m and 1 Hz was given. The latency of MEPs and area under curve (AUC) were used to evaluate the nerve conduction function of the rat hind limb. The shorter the latency of MEPs, the better the recovery of nerve function. The longer the area under curve, the better the recovery of nerve function.

### Catwalk gait analysis

Gait behavior and motor coordination were evaluated at 28 days after SCI (Hamers et al., 2006). The animals were allowed to walk on the bench several times before the experiment. The glass panel is then wiped clean and moistened with 75% alcohol which is sensitive to the signal and provides accurate data for analysis. The rats were then randomly placed in a gait tracking analysis system (Stones, China) to encourage the animals to walk in a straight line in order to obtain representative gait images for statistical analysis. The gait index and the average pressure intensity of hind limbs were statistically analyzed to evaluate the gait and motor function of rats.

### Determination of oxidative stress index (SOD, T-AOC, and MDA), SIRT1, and apoptosis index (Bcl2 and Bax) in SCI rats

In order to evaluate the changes of oxidative stress and cell apoptosis in the spinal cord microenvironment of SCI rats after H-sEVs treatment, rats at 7 days after surgery were randomly selected to be euthanized by inhalation of isoflurane (induced concentration 3%–5%). Then the heart was infused with cold saline to flush the blood away for sampling. Immediately afterwards, the spinal cord was exposed along the original surgical incision, and the spinal vertebrae were cut at two levels above and below the T10 segment with bone masseurs. The spinal tissue was carefully separated, and the blood on the spinal cord surface was rinsed with normal saline. Then the tissues were frozen with liquid nitrogen, and the prepared RIPA lysate (containing 1% PMSF, Beyotime, China) was added. The lysate tissues were fully ground on ice with a tissue grinding rod for 10 min. Then the tissue was thoroughly crushed by ultrasonic grinder (parameter: 100 w, 10 s/time, gap time: 10 s, repeated 3–5 times) and centrifuged in a centrifuge at 10,000 g, 4°C for 5 min. Part of the supernatant was used for the detection of oxidative stress and part was used for the detection of apoptosis. Strictly according to the SOD, T-AOC, and MDA kit (Nanjing Jiancheng, China) instructions for relevant tests. Apoptosis indicators and SIRT1 were detected by WB method, and the specific steps were as above.

### Nissl staining, H&E staining, SIRT1 immunohistochemical staining, and TUNEL immunofluorescence staining in SCI rats

At the corresponding time point, anesthesia was induced by inhalation of isoflurane (induction concentration 3%–5%) for euthanasia, followed by injection into the heart with cold saline and 10% formaldehyde for blood flushing and fixation. Immediately afterwards, the spinal cord was exposed along the original surgical incision, and the spinal vertebrae were cut at the upper and lower sections of the T10 segment with bone masher scissors. The spinal tissue was carefully separated, and the blood on the surface of the spinal cord was rinsed with normal saline and fixed with 10% formaldehyde. After dehydration, the tissue was embedded in paraffin and sliced for subsequent experiments. After the materials are collected, the animal carcasses shall be disposed harmlessly according to relevant regulations. Nissl staining and H&E staining (Beyotime, China) were performed strictly according to the procedure steps in the kit instructions to observe the survival of neurons and the size of the lesion area.

#### SIRT1 immunohistochemical staining

Tissue sections were dewaxed to water, then soaked in citrate and boiled for 20 min, and naturally cooled to room temperature for antigen repair. PBS containing 0.1% Triton X-100 was added and incubated at 4°C for 10 min to break the membrane. PBST was used to wash for 3 times and 5 min/each time. Then, 3% H_2_O_2_ was added to incubate at room temperature for 10 min to reduce the non-specific background staining caused by endogenous peroxidase. PBST was used to wash for 3 times and 5 min/each time. Then, 10% goat serum was added to incubate at room temperature for 30 min to seal the non-specific background staining. PBST was used to wash for 3 times and 5 min/each time. The primary antibody anti-SIRT1 was added by drops, overnight at 4 °C. PBST was used to wash for 3 times and 5 min/each time. The polymer adjuvant was added, and incubated at 37°C for 20 min. PBST was used to wash for 3 times and 5 min/each time. The enzyme-conjugate secondary antibody was added, and incubated at 37°C for 30 min. PBST was used to wash for 3 times and 5 min/each time. DAB color developer was added to the sample and color development was observed under a microscope (Leica, USA). The slide is then rinsed with running water to stop the reaction. Hematoxylin was redyed for 1–5 min and rinsed with running water for 1 min. The hydrochloric acid alcohol (1%) was used for differentiation for 20 s, and then tap water was used for rinsing for 1 min. Dilute ammonia water was used to return the blue for 30 s, and then tap water was used to rinse for 1 min. Finally, after gradient alcohol is used for dehydration, neutral gum is used for transparent seal. Results determination and analysis: SIRT1 expression was located in cells, and the staining was brown as positive cells. More than five high power fields (HPF) were randomly selected from the injury center and peripheral areas. Finally, ImageJ software (National Institutes of Health, Bethesda, MD, US) was used to analyze the Integral Optical Density (IOD) of selected visual fields.

#### TUNEL immunofluorescence staining

The apoptosis of spinal cord cells in each group was detected by TUNEL staining (Beyotime, China) after 7 days of SCI. The tissue sections were routinely dewaxed to water and washed with PBS for 5 min. The tissue was circled and PBS containing 0.1% Triton X-100 was added and incubated at 4 °C for 10 min to break the membrane. PBST was used to wash for 3 times and 5 min/each time. The appropriate amount of 3% hydrogen peroxide was added to cover the tissue, and incubated in a 37°C incubator for 20 min to eliminate endogenous peroxidase activity. An appropriate amount of 20 μg/mL protease K without DNase (Beyotime, China) was added to remove the interference of protease in tissues, and incubated at 37°C for 15 min. The TUNEL dye mixture was washed 3 times with PBS for 5 min each time and prepared according to the manufacturer’s instructions. 50 μL of TUNEL dye mixture was added to each sample and incubated for 1 h in a humidification chamber at 37°C away from light. Subsequently, the tablets were washed 3 times with PBS for 5 min each time, and sealed with anti-fluorescence quenching tablets containing DAPI (Beyotime, China) at room temperature and away from light. Six sections were randomly selected from each group and TUNEL positive cells were observed under a fluorescence microscope (Leica, Germany) on the injured area. Finally, the fluorescence intensity of TUNEL positive cells was analyzed by ImageJ software (National Institutes of Health, Bethesda, MD, US).

#### H-sEVs tracer *in vitro*

A bottle of P5 generation MSC cells at logarithmic growth stage was taken, trypsinized, washed twice with PBS, cell count was performed, cell density was adjusted to 1 × 10^6^ cells/mL, and the concentration of CFSE (MCE, USA) was added to 5 μM, and incubated in a 37°C incubator for 10 min. DMEM was added to complete culture medium, incubated at 37°C for 10 min to terminate the reaction, centrifuged at 300 g for 5 min, and cell precipitation was retained. The product was thoroughly washed 3 times with PBS to remove residual CFSE. Finally, the cells were re-suspended with 10% complete culture medium without exosome serum (Cyagen, China), re-inoculated into T75 culture flask, incubated in a hypoxic workstation (DWS, UK) for 48 h, and supernatant was collected. The supernatant collected above was separated to obtain H-sEVs with CFSE label according to the previous scheme. Highly differentiated PC12 neuron-like cells (Chinese Academy of Sciences, China) at logarithmic growth stage were selected, trypsinized and counted to adjust the cell concentration to 1 × 10^6^ cells/mL, and inoculated into a six-hole plate covered with polylysine coated cover glass. When PC12 cells grew to 50%–60% in complete culture medium, 100 μL CFSE-labeled H-sEVs (0.2 μg/μL) were added. After co-culture for 24 h, the supernatant of the cells was removed, washed in pre-cooled PBS for 2–3 times, and 4% paraformaldehyde was added and fixed at 4°C for 30 min, away from light. After washing with PBS for 3 times and 5 min/each time, 0.3% Triton was added to permeabilize the membrane at 4°C for 5 min. After washing with PBS for 3 times and 5 min/each time, 10% sheep serum was enclosed in room temperature for 1 h. After washing with PBS for 3 times and 5 min/each time, the cell creep tablets were carefully removed. DAPI anti-fluorescence quench agent (Beyotime, China) was added to the drops, and the tablets were sealed. The uptake of CFSE-labeled H-sEVs by PC12 cells was observed by fluorescence microscopy (Leica, Germany).

### Establishment and grouping of PC12 cell oxidative damage model induced by H_2_O_2_

Cells at logarithmic growth stage were selected, cell concentration was adjusted to 5 × 10^4^ cells/mL, and cell suspension of 100 μL per well was inoculated into 96-well plates. After cell adhesion, six experimental groups were set up, among which the normal control group: the same volume of complete medium was added for culture without any other treatment. H_2_O_2_-induced groups: Cells were stimulated with 100, 200, 400, 600, and 800 μM H_2_O_2_ (Aladdin, China), respectively. Each group had six wells and cultured in 37°C, 5% CO_2_ cell incubator. After 4 h of stimulation, the old medium was carefully sucked out. After washed with PBS for 3 times, the 100 μL complete medium and 10 μL CCK-8 reaction solution (Beyotime, China) were added to each well. After incubation at 37°C for 30 min, the light absorption value at 450 nm was measured with an enzyme label (Thermo Scientific, USA). Then the cell survival rate was calculated according to the light absorption value, and the optimal damage concentration of H_2_O_2_ was determined. The experiment was repeated three times.

PC12 cells at logarithmic growth stage were selected and the cell concentration was adjusted to 5 × 10^4^ cells/mL, and 2 mL cell suspension per well was inoculated into 12-well plates. After cell adhesion, four experimental groups were set up. NC group: Complete medium of the same volume was added for culture without any other treatment. H-sEVs group: Complete medium containing 100 μL H-sEVs (0.2 μg/μL) was added to the same volume for 4 h, and then changed to complete medium containing 100 μL H-sEVs (0.2 μg/μL) for 24 h H_2_O_2_ + PBS group: After being stimulated with complete medium containing 200 μM-H_2_O_2_ for 4 h, they were replaced with complete medium containing equal volume PBS for 24 h H_2_O_2_ + H-sEVs group: After 4 h stimulation with 200 μM-H_2_O_2_, the culture medium was changed to 100 μL H-sEVs (0.2 μg/μL) for 24 h.

### Determination of oxidative stress index and cell activity in PC12 cells

ROS testing is conducted in strict accordance with the ROS kit (Beyotime, China). After the cells were trypsinized and the concentration was adjusted to 5 × 10^4^ cells/mL, 2 mL per well was inoculated into the 12-well plate. After the cells were attached to the wall and treated according to the above grouping scheme, DMEM containing a dilution ratio of 1:1000 DCFH-DA was added to each well of the culture plate, and incubated in a CO_2_ incubator at 37°C for 20 min. The culture-medium was removed. After washed 3 times in pre-cooled PBS, the fresh complete culture-medium was added. Six visual fields were randomly selected under fluorescence microscope (Leica, Germany). Oxidative stress indicators (SOD, T-AOC, and MDA) were performed strictly according to the instructions provided by the reagent manufacturer. Cell activity was detected by CCK-8 method, as described above.

### Flow cytometry of annexin V FITC/PI apoptosis staining in PC12 cells

The cells were treated according to the grouping scheme described above. The cells were trypsinized and collected, and washed twice with pre-cooled PBS. According to the instructions of Annexin V FITC/PI apoptosis staining kit (Beyotime, China), cells were re-suspended with 1× binding buffer, and 1 μL Annexin V and 5 μL PI dyes were added under dark condition. The cells were mixed and incubated at room temperature and dark for 5 min. C6 flow cytometry (BD, USA) was used for machine detection. The percentage of apoptotic cells in all cells was the apoptosis rate of PC12 cells.

### Oxidative stress proteins (SIRT1, Nrf2, and HO-1), apoptosis-related proteins (Bcl2 and Bax) and internal reference proteins (β-actin) of PC12 cells were detected by WB assay

The cells were treated according to the grouping scheme described above. After PC12 cells were collected, RIPA lysate (containing 1% PMSF, Beyotime, China) was added and mixed, then quickly and repeatedly blown on ice for 1 min and left for 10 min. After the nucleic acid was broken by ultrasonic, the samples were centrifuged at 4°C, 12,000 g, for 10 min. The precipitation was abandoned, and the supernatant was taken. The protein concentration was determined by BCA kit (Beyotime, China). Then the supernatant was added a loading buffer with a volume ratio of 1:4 to adjust the concentration uniformly. The samples with the same concentration were heated in boiling water at 100°C for 5 min to denaturate, and then 20 μg protein and marker were added with prefabricated glue. Then electrophoresis was performed at constant pressure 160 V for 40 min. After electrophoresis, the proteins were transferred to the NC membrane using a sandwich method at 110 V wet for 1 h (the time was adjusted according to the molecular weight of the specific target protein). They were incubated in a shaker at room temperature for 2 h using a closed solution (5% skim milk powder). Primary antibodies anti-SIRT1 (Cat# sc-74465, Santa Cruz Biotechnology), anti-Nrf2 (Cat# PA5-27882, ThermoFisher Scientific), anti-HO-1 (Cat# ab68477, Abcam), anti-Bcl-2 (Cat# ab196495, Abcam), anti-Bax (Cat# ab196495, Abcam), and anti-β-actin (Cat# MA1-140, ThermoFisher Scientific) were added and incubated at 4°C overnight. On the second day, primary antibodies were discarded, and TBST was used to wash for 3 times and 5 min/each time. After incubation with the corresponding secondary antibody at room temperature for 1 h, the membrane was washed with TBST for 3 times, 10 min each time. Finally, using the enhanced ECL kit (Beyotime, China), the developing droplets were prepared and added to the front of the NC membrane. After reacting for several seconds, the droplets were exposed under the e-Blot gel imager (e-BLOT, China).

### PC12 cell lentivirus transfection inhibited SIRT1 expression

PC12 cells at logarithmic growth stage were trypsinized, and cell count was performed. The cell concentration was adjusted to 1 × 10^5^ cells/mL, and the cells were inoculated into 24-well plates. After cell adherence, the original medium was replaced with a medium containing 5 μg/mL polybrene, and lentiviral vectors (LV-SIRT1-IN-GFP group) with GFP fluorescence labeling (GenePharma, China) were added (sequence number: 5′-GAG​GCA​GTT​AAT​GAA​GCT​ATA-3′) and appropriate viral vector suspension (LV-NC group). After incubating at 37 °C for 24 h, replace the fresh medium. The culture was continued for 48 h, cleaned 3 times with PBS, and then replaced with fresh medium. The single-digit cells were trypsinized and inoculated into 96-well plates for several times, observed by fluorescence microscope (Leica, Germany), and the GFP labeled cells were screened repeatedly to ensure a high knockout rate. Finally, fluorescence microscopy (Leica, Germany) and C6 flow cytometry (BD, USA) were used to observe the knockout efficiency. Meanwhile, cell proteins were extracted and WB was used to verify the knockout efficiency.

### Statistical analysis

Statistical data were expressed as mean ± standard deviation (SD) and analyzed statistically by GraphPad Prism 8.0 software (La Jolla, CA, USA). All experiments were repeated three times. One-way ANOVA analysis and two-way ANOVA analysis were used to compare the differences between multiple groups, and independent sample T-test was used to compare the differences between two groups. *p*-values <0.05 indicated statistically significant differences.

## Results

### Identification of MSCs and H-sEVs

Under optical microscope (Supplementary Figure S2A), the primary cultured MSCs of P5 generation showed adherent growth, vorticular distribution, long spindle shape, long protrusions at both ends, and oval and centered nuclei. CD29 and CD90 were positive markers of MSCs, while CD45 and CD34 were negative markers of MSCs. Flow cytometry (Supplementary Figure S2B) showed that 98.4% and 99.5% of CD29 and CD90 cells had positive surface markers, both higher than 95%, but 1.9% and 1.3% of CD45 and CD34 cells had positive surface markers, both lower than 5%. Alizarin red, oil red O, and alcian blue staining showed microscopic deposits of red calcium nodules, red fat droplets, and intracellular blue acid mucosaccharides. These results indicated that MSCs of P5 generation differentiated into osteoblasts, adipocytes, and chondrocytes (Supplementary Figure S2C). In conclusion, MSCs were successfully isolated and cultured from rat bone marrow. According to protocol recommended by the International Extracellular Vesicle Association (Thery et al., 2018), the following three methods were selected for the identification of H-sEVs, including TEM, NTA, and WB. TEM observation (Supplementary Figure S3A) showed that H-sEVs had circular or quasi-circular membranous structures, and were cupped like typical bilayer membranes, with a median diameter of 120 nm, which was consistent with the characteristics of H-sEVs under electron microscopy. It could be seen from the NTA results (Supplementary Figure S3B) that the diameter of H-sEVs was scattered in a normal distribution, with a diameter distribution of 30–200 nm and a concentration of 7.6E+10 particles/mL. WB results showed that positive marker proteins CD9, CD63, and TSG101 were expressed in H-sEVs (Supplementary Figure S3C), and the expression levels were higher than those of MSCs (Supplementary Figure S3D). In conclusion, we can successfully separate and purify H-sEVs by density gradient ultracentrifugation.

### H-sEVs promote functional recovery of hind limbs in SCI rats

**FIGURE 1:**
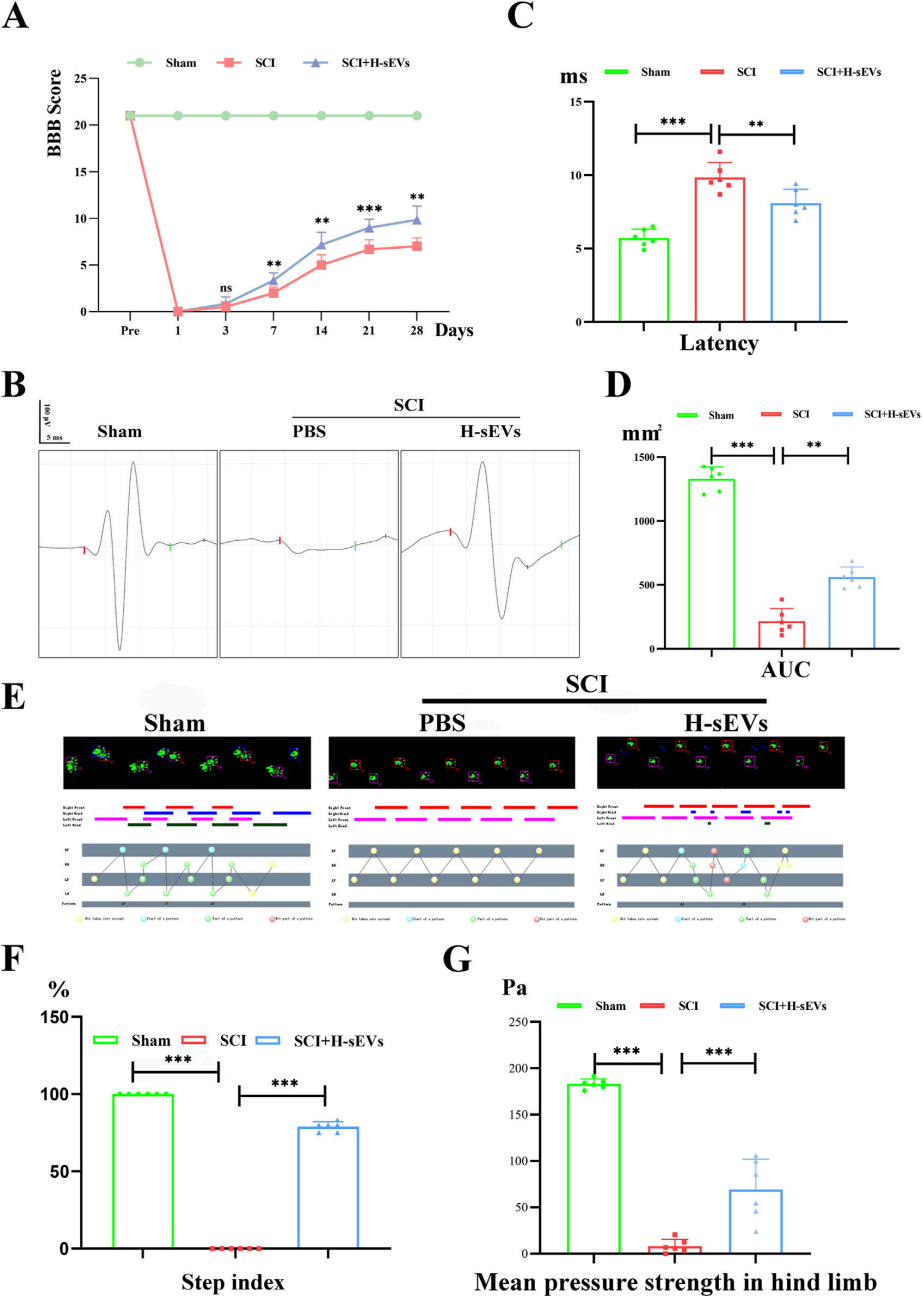
H-sEVs promote recovery of motor function in SCI rats. **(A)** BBB score (1 day before operation, and at 1, 3, 7, 14, 21, and 28 days after operation). **(B)** Electrophysiological monitoring at 28 days after operation. The time from the beginning to the red bar marks the latency. The area under the curve from the red bar to the green bar represents the AUC. **(C, D)** Statistical analysis of latency and AUC. **(E)** Catwalk analysis at 28 days after operation. **(F, G)** Statistical analysis of step index and mean pressure in hind limb. (n = 6, *p < 0.05, **p < 0.01, ***p < 0.001, ns: not significant).

The experimental process was basically stable and the baseline was consistent (Supplementary Figure S1). Among them, a total of five rats died due to excessive anesthesia during modeling and septic shock after modeling, and the number of samples was supplemented according to the corresponding treatment group. BBB score of SCI rats was performed by double-blind method (Figure 1A). It was found that both hind limbs of SCI group and H-sEVs group showed no obvious movement on the first day after injury, mainly relying on forelimbs dragging and crawling, which was 0 point. On day 3 after injury, there was no difference between the SCI group and the H-sEVs group. On day 7 after injury, the motor function of both hind limbs in H-sEVs group was significantly improved compared with SCI group, and the average BBB score was 3.33 and 2.0 points, respectively (*p* < 0.01). With continued observation, the mean score of the H-sEVs group and SCI group was 7.17 and 5.0 on day 14 after injury, respectively (*p* < 0.01). On day 21 after injury, the mean score of the H-sEVs group was 9.0, significantly higher than that of the SCI group, which was 6.67 (*p* < 0.001). The functional scores of SCI group and H-sEVs group at 28 days were 7.33 and 9.83, respectively (*p* < 0.01). These results indicated that BBB motor function scores in H-sEVs group were significantly improved compared with SCI group at days 7, 14, 21, and 28 after injury. To further determine the therapeutic effect of H-sEVs on post-SCI neurological recovery, electrophysiological monitoring was performed at 28 days post-SCI (Figure 1B). MEP results showed that the latency of rats in the H-sEVs group at 28 days post-SCI was significantly reduced (Figure 1C, *p* < 0.01), and the AUC was significantly increased (Figure 1D, *p* < 0.01) compared with those in the SCI group. Finally, we also analyzed the hind limb coordination and motor function of SCI rats in each group at 28 days post-SCI using the Catwalk gait analysis system (Figure 1E). Step index reflects the motor coordination of rats, and the average pressure strength of hind limbs touching the ground reflects the motor function of hind limbs. The results showed that after 28 days of treatment with H-sEVs, the hind limbs were obviously touched on the sole of the foot, and the step index reached 80%, which was significant compared with the SCI group (Figure 1F, *p* < 0.001). The average pressure strength of the hind limbs in the H-sEVs group was significantly increased compared with that of the SCI group (Figure 1G, *p* < 0.001). These results indicate that H-sEVs can promote the recovery of hind limb motor function in SCI rats.

### H-sEVs alleviate oxidative stress in microenvironment and protect neurons after SCI

**FIGURE 2:**
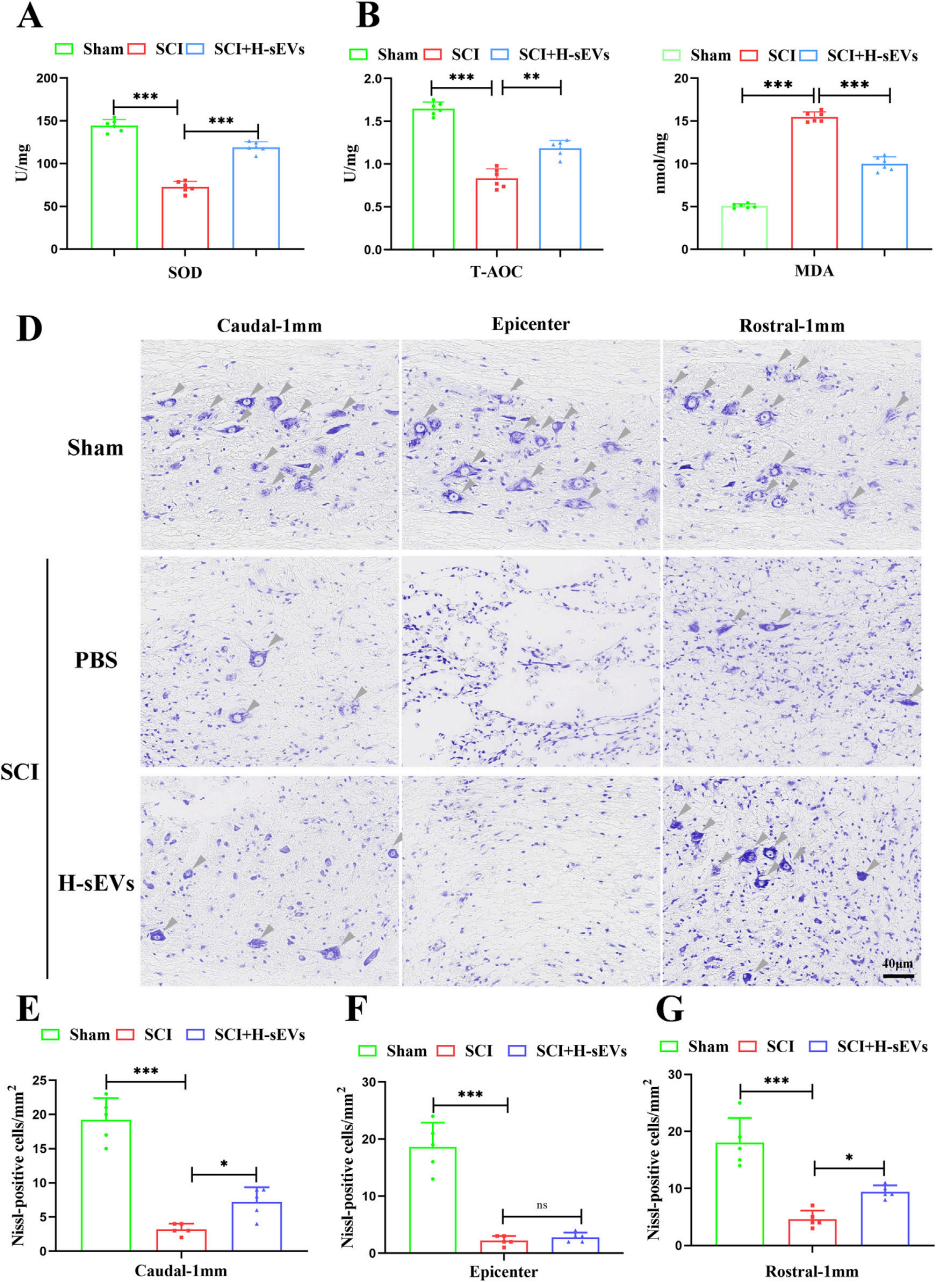
H-sEVs reduce oxidative stress and protect neurons after SCI. **(A–C)** The expression levels of SOD, T-AOC, and MDA in spinal cord tissue (n = 6). **(D)** Representative images of Nissl staining (Caudal-1mm, epicenter, and rostral-1mm, 20×, Scale bar: 40 μm, gray triangular arrow: neuron). **(E–G)** Statistical analysis of the number of neurons/mm^2^ at Caudal-1mm, epicenter, and rostral-1mm (n = 5). (*p < 0.05, **p < 0.01, ***p < 0.001, ns: not significant).

Oxidative stress is one of the main characteristics of spinal microenvironment changes after SCI. To evaluate the effect of H-sEVs treatment on oxidative stress in microenvironment after SCI. The indexes of oxidative stress (SOD, T-AOC, and MDA) in the spinal microenvironment of SCI rats in each group on day 7 were detected. Compared with SCI group, SOD (Figure 2A, *p* < 0.001) and T-AOC (Figure 2B, *p* < 0.01) contents in the spinal cord of H-sEVs group were significantly increased, while MDA (Figure 2C, *p* < 0.001) contents were significantly decreased. These results indicated that H-sEVs therapy could improve the antioxidant stress ability of SCI microenvironment in rats. Subsequently, Nissl staining was performed to observe the survival of neurons in the injury center and peripheral areas (Figure 2D). The results showed that only sporadic neuronal survival was observed in the injured center area after H-sEVs treatment, with no significant difference compared with SCI group (Figure 2F, *p* = 0.848). However, at the caudal (Figure 2E, *p* < 0.05) and the rostral (Figure 2G, *p* < 0.05) 1 mm from the epicenter, a higher number of neurons survived in the H-sEVs group than that in the SCI group. These results suggest that H-sEVs can protect neurons by improving oxidative stress in the tissue microenvironment after SCI.

### H-sEVs promote SIRT1 expression and spinal cord tissue repair after SCI

**FIGURE 3:**
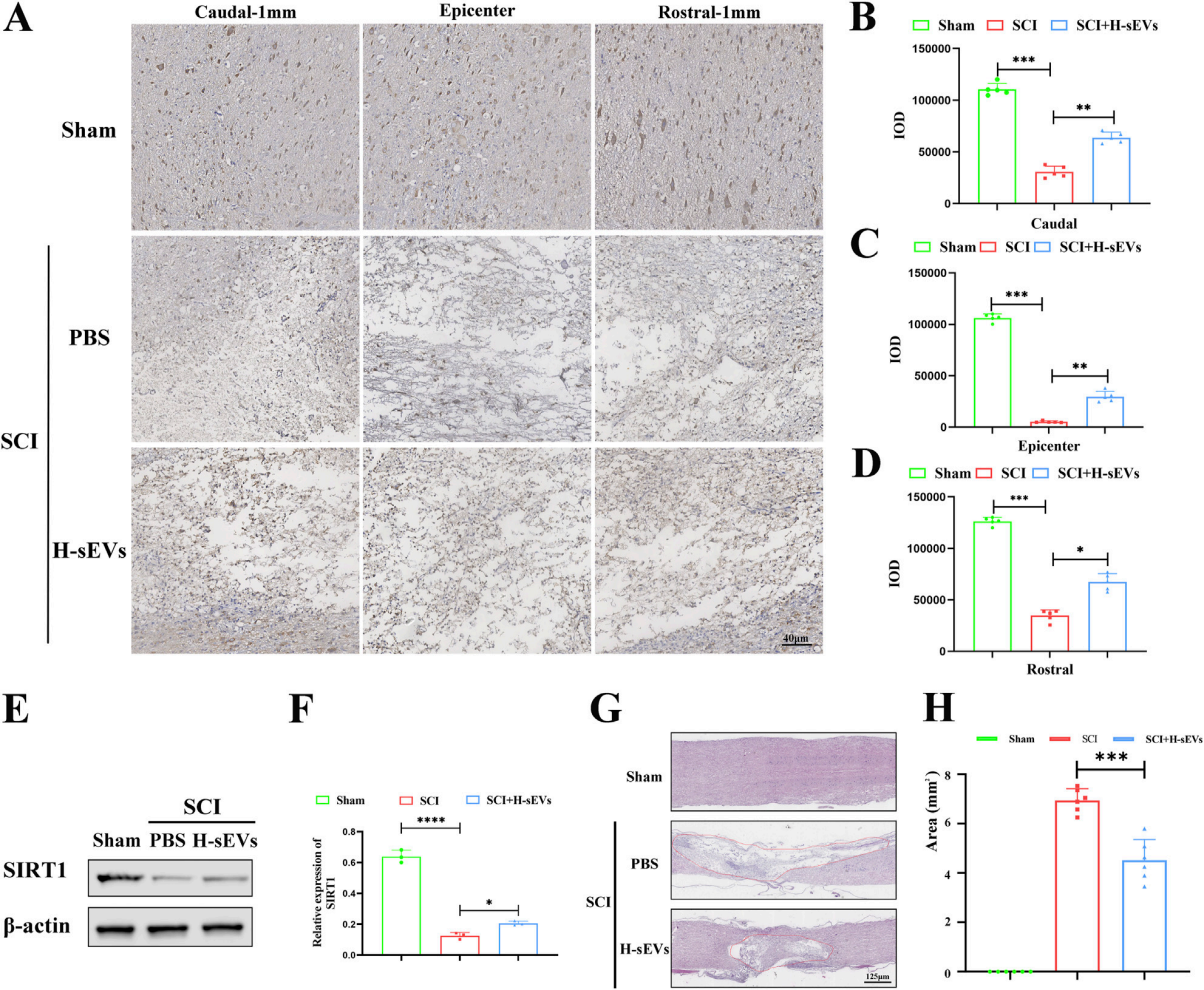
H-sEVs promote SIRT1 expression after SCI and spinal cord repair. **(A)** Representative immunohistochemical image of SIRT1 expression in the caudal-1mm, epicenter, and rostral-1mm of spinal cord tissue. **(B–D)** Statistical analysis of SIRT1 expression in the caudal-1mm, epicenter, and rostral-1mm of spinal cord tissue (n = 5). **(E)** Representative WB images in spinal cord tissue (SIRT1, internal reference protein: β-actin). **(F)** Statistical analysis of relative expression of SIRT1 in spinal cord tissue (n = 3). **(G)** HE staining of spinal cord tissue (red solid line marks the area of injury). **(H)** Statistical analysis of injury area in spinal cord tissue (n = 6). (*p < 0.05, **p < 0.01, ***p < 0.001, ns: not significant).

To determine whether H-sEVs can regulate SIRT1 and repair spinal cord tissue after SCI, the expression of SIRT1 in the spinal cord of rats in each group on day 7 after SCI was detected by immunohistochemistry (Figure 3A). We found that SIRT1 increased significantly in the lesion center after H-sEVs treatment, which was significantly different from that in the SCI group (Figure 3C, *p* < 0.01). At the same time, the expression of SIRT1 in the caudal (Figure 3B, *p* < 0.01) and the rostral (Figure 3D, *p* < 0.01) 1 mm from the epicenter was significantly increased in the H-sEVs group, which was significantly different from that in the SCI group. WB was used to analyze the expressions of SIRT1 protein *in vivo* (Figure 3E). The results showed that, compared with the SCI group, the expression level of SIRT1 was significantly increased in the H-sEVs group after injury (Figure 3F). The results showed that H-sEVs could significantly increase the expression level of SIRT1 in the spinal cord of SCI rats. Furthermore, H&E staining was performed on spinal tissue samples taken at day 28 after SCI (Figure 3G). The results showed that, compared with SCI group, the spinal cord tissue structure of rats treated with H-sEVs was significantly improved, the injury cavity was smaller, and the lesion area was significantly reduced (Figure 3H, *p* < 0.001). These results suggest that SIRT1 may be a key protein in promoting SCI repair by alleviating oxidative stress response of H-sEVs.

### H-sEVs alleviate apoptosis after SCI

**FIGURE 4:**
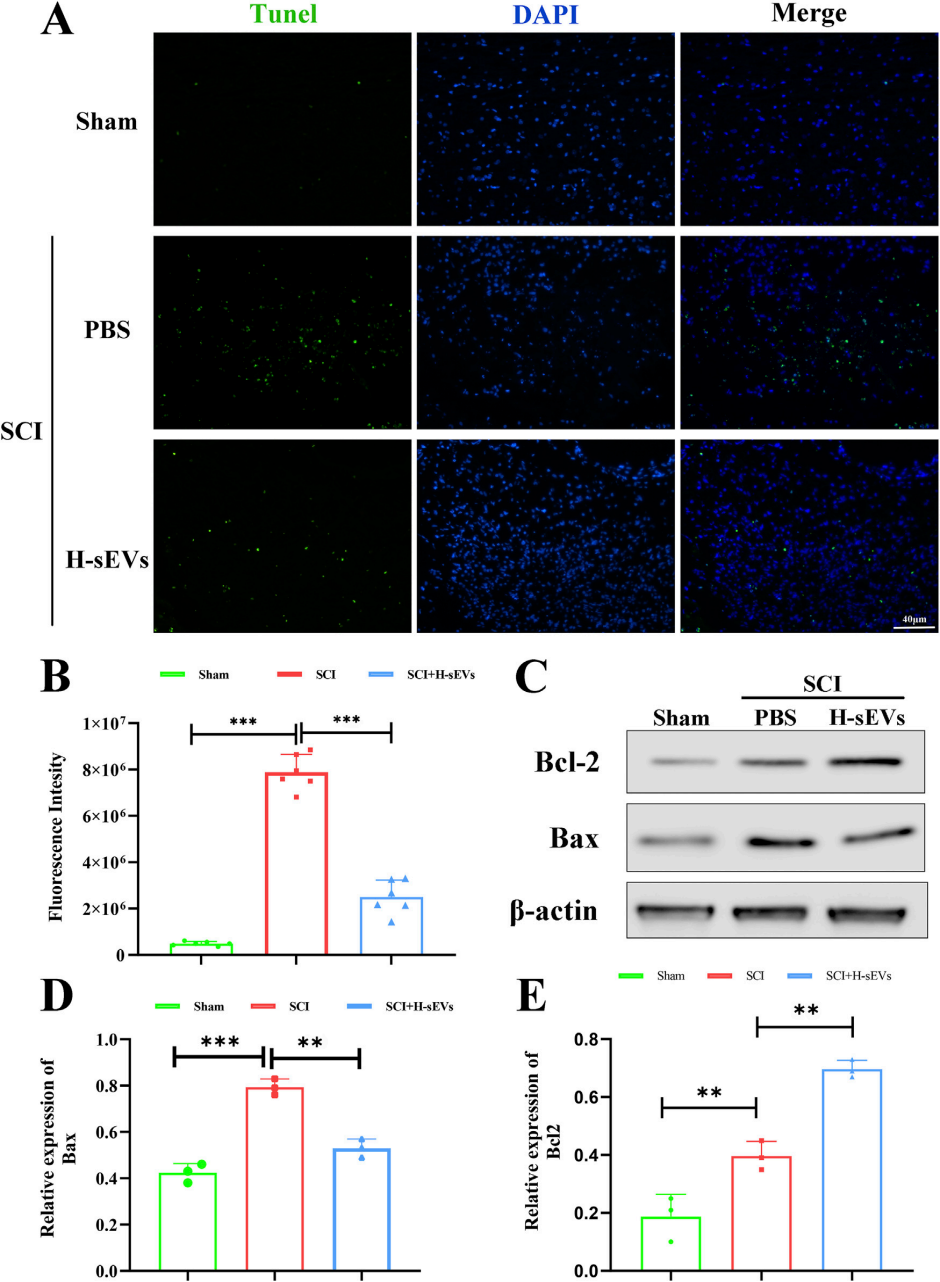
H-sEVs inhibit apoptosis after SCI. **(A)** Representative Tunel staining images of spinal cord tissue (green fluorescence represents apoptotic cells, blue fluorescence represents nuclei, 20×, Scale bar: 40 μm). **(B)** Statistical analysis of the fluorescence intensity of apoptotic cells (n = 6). **(C)** Representative WB images in spinal cord tissue (Bax and Bcl-2, internal reference protein: β-actin). **(D, E)** Statistical analysis of relative expression of Bax and Bcl-2 in spinal cord tissue (n = 3). (*p < 0.05, **p < 0.01, ***p < 0.001, ns: not significant).

To determine whether administration of H-sEVs alleviates apoptosis in spinal cord tissue after SCI, the TUNEL staining was performed on spinal tissue sections at day 7 post-SCI (Figure 4A). We found that the number of apoptotic cells in the spinal cord was significantly lower in the H-sEVs group than that in the SCI group (Figure 4B, *p* < 0.001). Subsequently, we also extracted spinal cord protein on day 7 after SCI, and WB was used to analyze the expressions of anti-apoptotic protein Bcl-2 and pro-apoptotic protein Bax *in vivo* (Figure 4C). The results showed that, compared with the SCI group, the expression level of Bax was significantly decreased in the H-sEVs group after injury (Figure 4D), while the expression level of Bcl-2 was significantly increased (Figure 4E). These results suggest that H-sEVs can alleviate apoptosis after SCI.

### H-sEVs attenuate H_2_O_2_-induced apoptosis of PC12 cells

**FIGURE 5:**
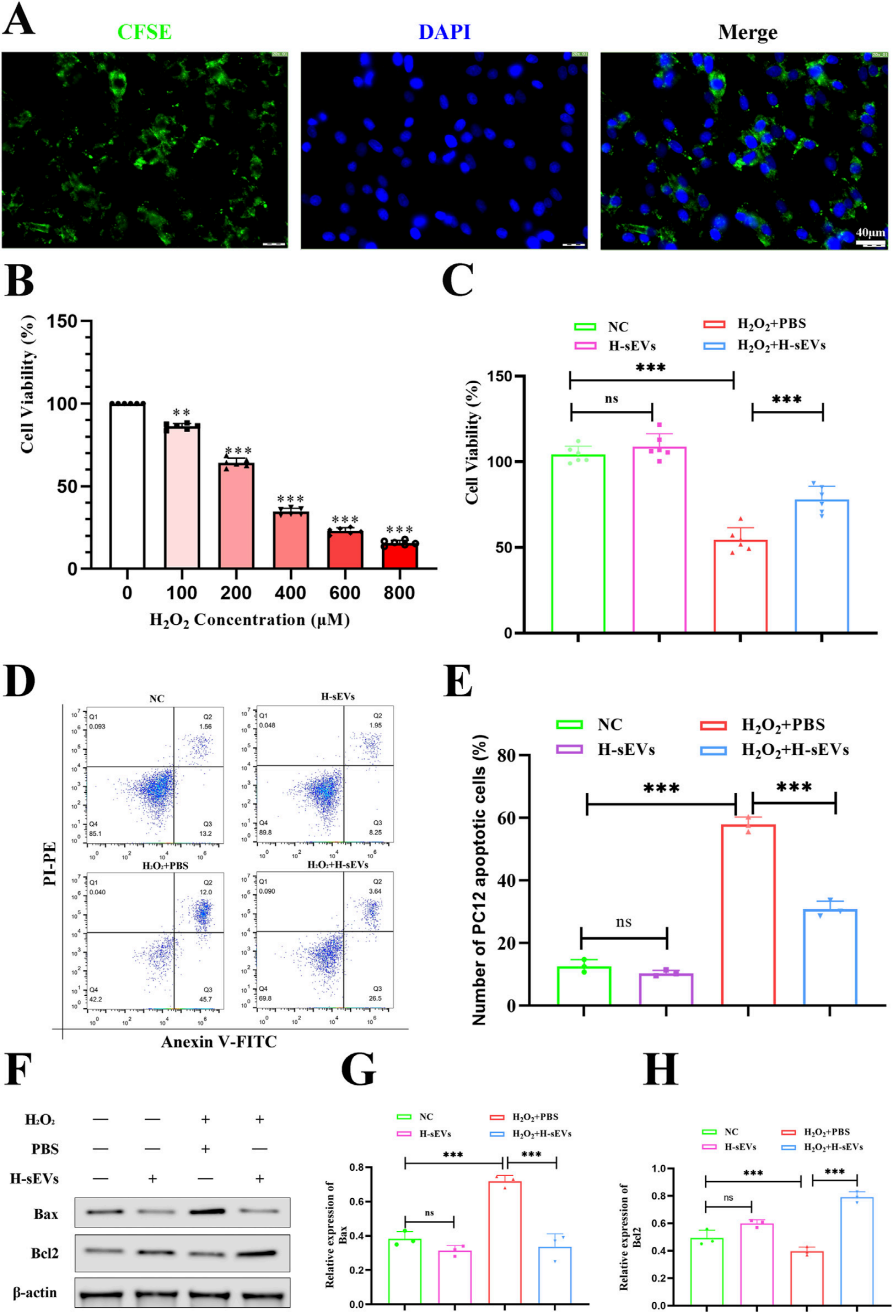
H-sEVs inhibit H_2_O_2_-induced apoptosis in PC12 cells. **(A)** Phagocytosis of CFSE-labeled H-sEVs by PC12 cells under fluorescence microscopy (green fluorescence represents CFSE-labeled H-sEVs, blue fluorescence represents nuclei, 20×, Scale bar: 40 μm). **(B)** Cell activity after 4 h stimulation of PC12 with H_2_O_2_ at different concentrations. **(C)** Cell viability measured by CCK-8 assay in different group of PC12 cells (n = 6). **(D)** Representative flow analysis image of apoptosis in different group of PC12 cells by Annexin V FITC/PI apoptosis assay. **(E)** Statistical analysis of PC12 cell apoptosis ratio (n = 3). **(F)** Representative WB images of Bax and Bcl-2 in PC12 cells (internal reference protein: β-actin). **(G, H)** Statistical analysis of relative expression of Bax and Bcl-2 in PC12 cells (n = 3). (*p < 0.05, **p < 0.01, ***p < 0.001, ns: not significant).

First, in order to observe whether PC12 cells can phagocytic H-sEVs, we used CFSE-labeled MSCs, cultured them in a hypoxic environment, and isolated and purified CFSE-labeled H-sEVs according to the previous methods. CFSE-labeled H-sEVs were added into PC12 cell culture dishes, incubated for 24 h, and cell slides were prepared. Under fluorescence microscope, a large number of scattered granular green fluorescence could be seen in the cytoplasm of PC12 cells, indicating that CFSE-labeled H-sEVs could be taken up by PC12 cells (Figure 5A). Second, in order to find the optimal stimulation concentration of H_2_O_2_ to damage PC12 cells, different concentrations of H_2_O_2_ were given to stimulate PC12 cells, and CCK-8 detection kit was detected after 4 h (Figure 5B). We found that the survival rate of PC12 cells was negatively correlated with H_2_O_2_ concentration. With the increase of H_2_O_2_ stimulation concentration, the cell survival rate gradually decreased. When H_2_O_2_ concentration reached 200 μM, the cell survival rate was about 65% (*p* < 0.001). At this point, PC12 cells are damaged to a certain extent, but they are still in a reversible state. Therefore, the 200 μM-H_2_O_2_ for 4 h was selected as the best stimulation condition for PC12 cells. Then, in order to investigate whether H-sEVs can improve the cell activity and apoptosis of PC12 cells stimulated by H_2_O_2_. We measured the activity of PC12 cells using CCK-8 detection kit (Figure 5C). The results showed that compared with the NC group, there was no significant difference in the proliferation ability of PC12 cells treated with H-sEVs without H_2_O_2_ stimulation. However, cell activity was significantly decreased in the H_2_O_2_ + PBS group compared with the NC group (*p* < 0.001). Compared with H_2_O_2_ + PBS group, the cell activity of PC12 cells was significantly improved after H_2_O_2_ stimulation by H-sEVs co-culture (*p* < 0.001). Subsequently, flow cytometry was used to determine the apoptosis of PC12 cells in different treatments (Figure 5D). The results showed that there was no statistical difference in the number of apoptosis in the H-sEVs group compared with the NC group. However, compared with the NC group, the number of apoptotic cells was significantly increased in the H_2_O_2_ + PBS group (Figure 5E, *p* < 0.001). The number of apoptotic cells in the H_2_O_2_ + H-sEVs group was significantly lower than that in the H_2_O_2_ + PBS group (Figure 5E, *p* < 0.001). Finally, proteins from PC12 cells were extracted and WB was used to verify the expressions of apoptosis-related proteins Bcl-2 and Bax (Figure 5F). The results showed that the apoptosis-related proteins Bcl-2 and Bax in the H-sEVs group had no significant changes compared with the NC group. However, compared with NC group, anti-apoptotic protein Bcl-2 were significantly decreased and pro-apoptotic protein Bax were significantly increased in H_2_O_2_ + PBS group (Figures 5G, H, *p* < 0.001). Compared with H_2_O_2_ + PBS group, anti-apoptotic protein Bcl-2 were significantly increased and pro-apoptotic protein Bax were significantly decreased in H_2_O_2_ + H-sEVs group (Figures 5G, H, *p* < 0.001). These results indicated that H-sEVs treatment had no significant effect on the cell activity and apoptosis of normal and undamaged PC12 cells. For PC12 cells after H_2_O_2_-induced injury, H-sEVs treatment can significantly improve cell activity and inhibit cell apoptosis, so as to play a role in cell protection.

### H-sEVs alleviate oxidative stress in H_2_O_2_-induced PC12 cells

**FIGURE 6:**
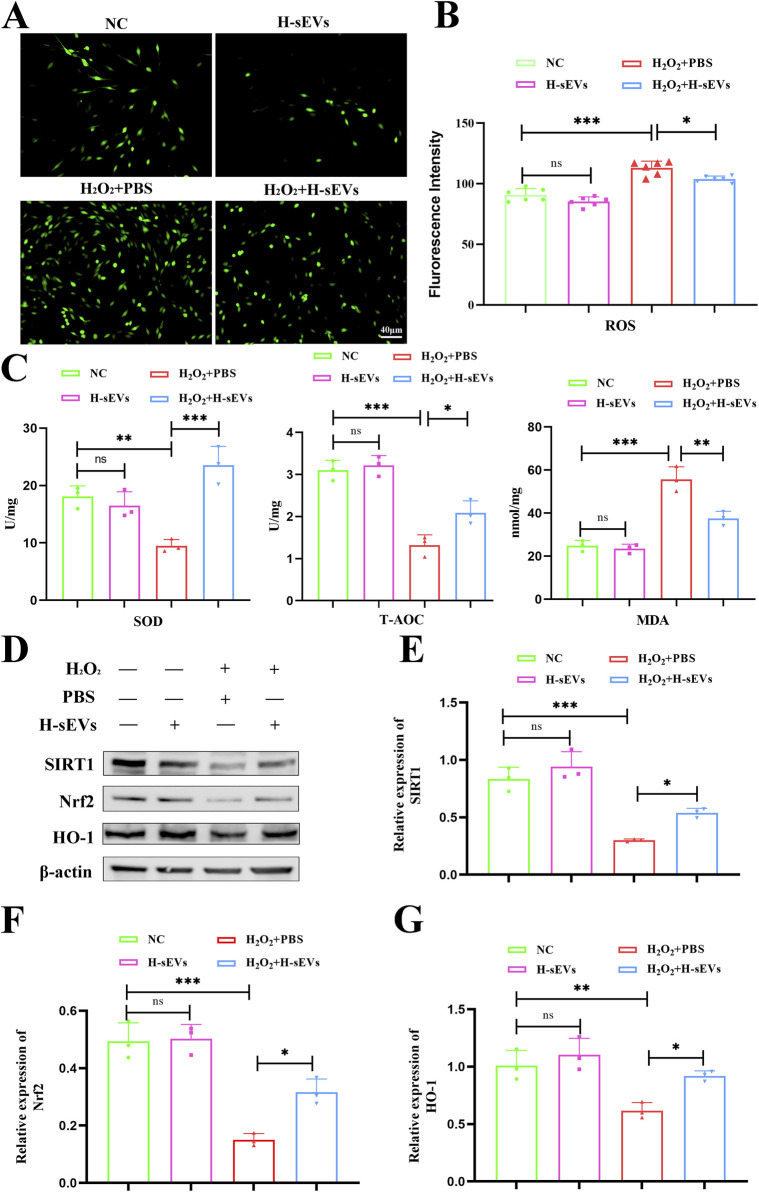
H-sEVs alleviate oxidative stress in H_2_O_2_-induced PC12 cells. **(A)** ROS representative images of different groups of PC12 cells under fluorescence microscopy (green fluorescence represents ROS, 20×, Scale bar: 40 μm). **(B)** Statistical analysis of mean fluorescence intensity of ROS expression in PC12 cells (n = 6). **(C)** The expression levels of SOD, MDA, and T-AOC in different groups of PC12 cells (n = 3). **(D)** Representative WB images of SIRT1, Nrf2, and HO-1 in different group of PC12 cells (internal reference protein: β-actin). **(E–G)** Statistical analysis of relative expression of SIRT1, Nrf2, and HO-1 in different group of PC12 cells (n = 3). (*p < 0.05, **p < 0.01, ***p < 0.001, ns: not significant).

Oxidative stress reaction is one of the key factors of microenvironment homeostasis imbalance after SCI. In order to investigate whether H-sEVs could alleviate H_2_O_2_-induced oxidative damage in PC12 cells, ROS expression levels in PC12 cells treated with different treatments were detected using ROS detection kit, and then observed under fluorescence microscope (Figure 6A). The results showed that ROS expression level of PC12 cells did not change significantly after co-culture with H-sEVs alone compared with NC group. However, ROS expression levels were significantly higher in the H_2_O_2_ + PBS group than that in the NC group (Figure 6B, *p* < 0.001). Compared with H_2_O_2_ + PBS group, ROS expression levels in H2O2+H-sEVs group were significantly decreased (Figure 6B, *p* < 0.05). Meanwhile, SOD, T-AOC, and MDA of PC12 cells in each group were detected (Figure 6C). The results showed that, compared with NC group, the oxidative stress indexes (SOD, T-AOC, and MDA) of H-sEVs group had no significant changes. However, compared with NC group, the expression level of oxidation index MDA was significantly increased in H_2_O_2_ + PBS group (*p* < 0.001), and the expression levels of antioxidant indexes SOD (*p* < 0.01) and T-AOC (*p* < 0.001) were significantly decreased in H_2_O_2_ + PBS group. Compared with H_2_O_2_ + PBS group, the expression level of oxidation index MDA in H_2_O_2_ + H-sEVs group was significantly decreased (*p* < 0.01), and the expression levels of antioxidant indexes SOD (*p* < 0.001) and T-AOC (*p* < 0.05) were significantly increased. Subsequently, proteins of PC12 cells in each group were extracted and WB method was used to verify the expressions of oxidative stress-related proteins SIRT1, Nrf2, and HO-1 (Figure 6D). The results showed that the oxidative stress-related proteins SIRT1, Nrf2, and HO-1 had no significant changes in the H-sEVs group compared with the NC group. However, compared with NC group, antioxidant stress proteins SIRT1 (Figure 6E, *p* < 0.001), Nrf2 (Figure 6F, *p* < 0.001), and HO-1 (Figure 6G, *p* < 0.01) were significantly decreased in H_2_O_2_ + PBS group. Compared with H_2_O_2_ + PBS group, the antioxidant stress proteins SIRT1 (Figure 6E, *p* < 0.05), Nrf2 (Figure 6F, *p* < 0.05), and HO-1 (Figure 6G, *p* < 0.05) in H_2_O_2_ + H-sEVs group were significantly increased. In conclusion, H-sEVs treatment had no significant effect on the oxidative stress response of normal and undamaged PC12 cells. For PC12 cells after H_2_O_2_-induced injury, treatment with H-sEVs can significantly relieve the oxidative damage.

### SIRT1 is a key target of H-sEVs inhibition of neuronal death

**FIGURE 7:**
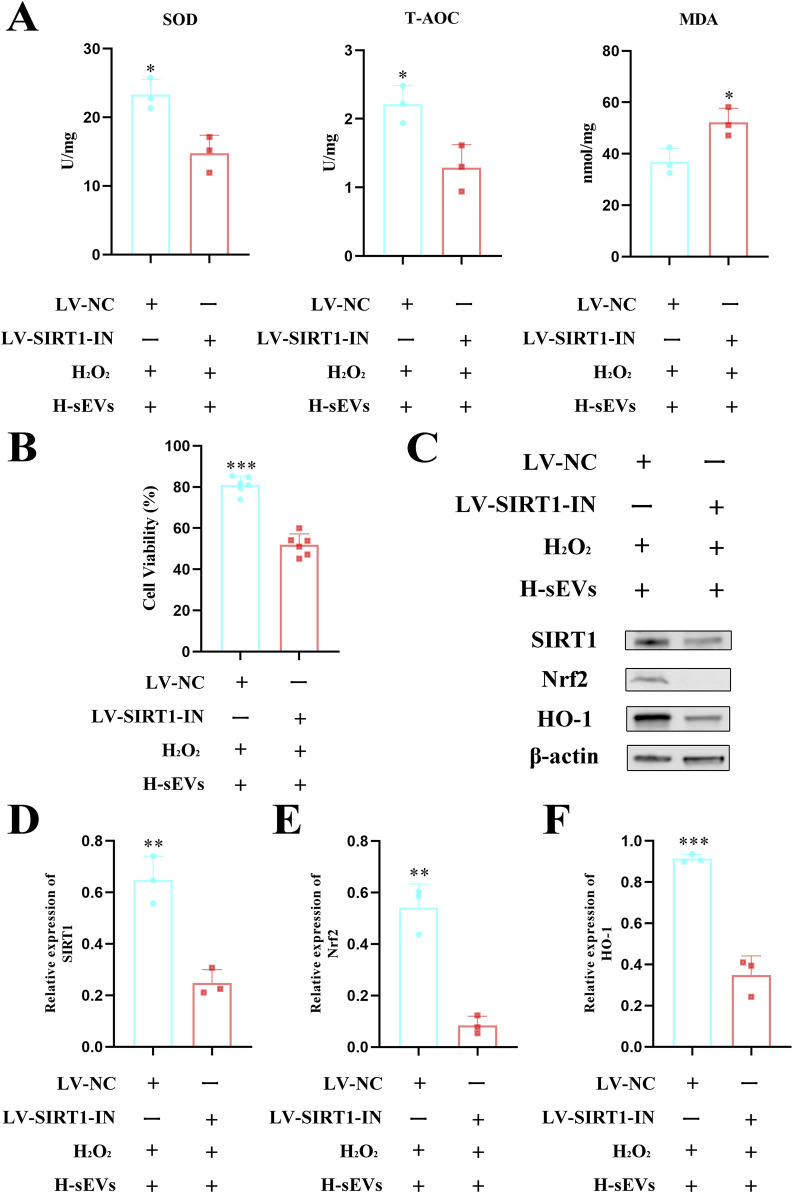
SIRT1 is a key target of H-sEVs inhibition of neuronal death. **(A)** The expression levels of SOD, T-AOC, and MDA in different groups of PC12 cells (n = 3). **(B)** Cell viability measured by CCK-8 assay in different group of PC12 cells (n = 6). **(C)** Representative WB images of SIRT1, Nrf2, and HO-1 in different group of PC12 cells (internal reference protein: β-actin). **(D–F)** Statistical analysis of relative expression of SIRT1, Nrf2, and HO-1 in different group of PC12 cells (n = 3). (*p < 0.05, **p < 0.01, ***p < 0.001, ns: not significant).

In order to further investigate the specific mechanism of H-sEVs alleviating oxidative stress response of neurons, we suppressed SIRT1 expression by transfecting PC12 cells with lentivirus. Fluorescence microscopy, flow cytometry, and WB were used to verify the knockout efficiency of SIRT1 (Supplementary Figure S4). The results showed that the knockout rate reached more than 85%. Subsequently, H_2_O_2_ was first used to stimulate PC12 cells with or without SIRT1 knockout to cause damage, and then H-sEVs were administered. Finally, the expression levels of SOD, T-AOC, and MDA (Figure 7A) and cell activity (Figure 7B) were detected in each group. Meanwhile, its cell proteins were extracted and WB was used to determine the expression levels of SIRT1 and its downstream proteins Nrf2 and HO-1 (Figure 7C). We found that compared with PC12 cells without SIRT1 knockout, the expression levels of antioxidant stress index SOD and T-AOC in PC12 cells with SIRT1 knockout were significantly decreased (*p* < 0.05), and the expression levels of oxidation index MDA were significantly increased (*p* < 0.05). Moreover, compared with PC12 cells without SIRT1 knockout, the cell activity of PC12 cells with SIRT1 knockout (*p* < 0.001), SIRT1 (Figure 7D, *p* < 0.01) and its downstream proteins Nrf2 (Figure 7E, *p* < 0.01) and HO-1 (Figure 7F, *p* < 0.001) were significantly reduced. In conclusion, after SIRT1 knockout in PC12 cells, H_2_O_2_-induced damage and then H-sEVs treatment significantly reduced the anti-oxidative stress efficacy of H-sEVs. These results indicate that H-sEVs play an anti-oxidative stress role by increasing the expression of SIRT1 in PC12 cells. Even, the expression levels of downstream proteins Nrf2 and HO-1 are significantly decreased, suggesting that H-sEVs may relieve oxidative stress damage of neurons through SIRT1/Nrf2/HO-1 signaling pathway.

## Discussion

**FIGURE 8:**
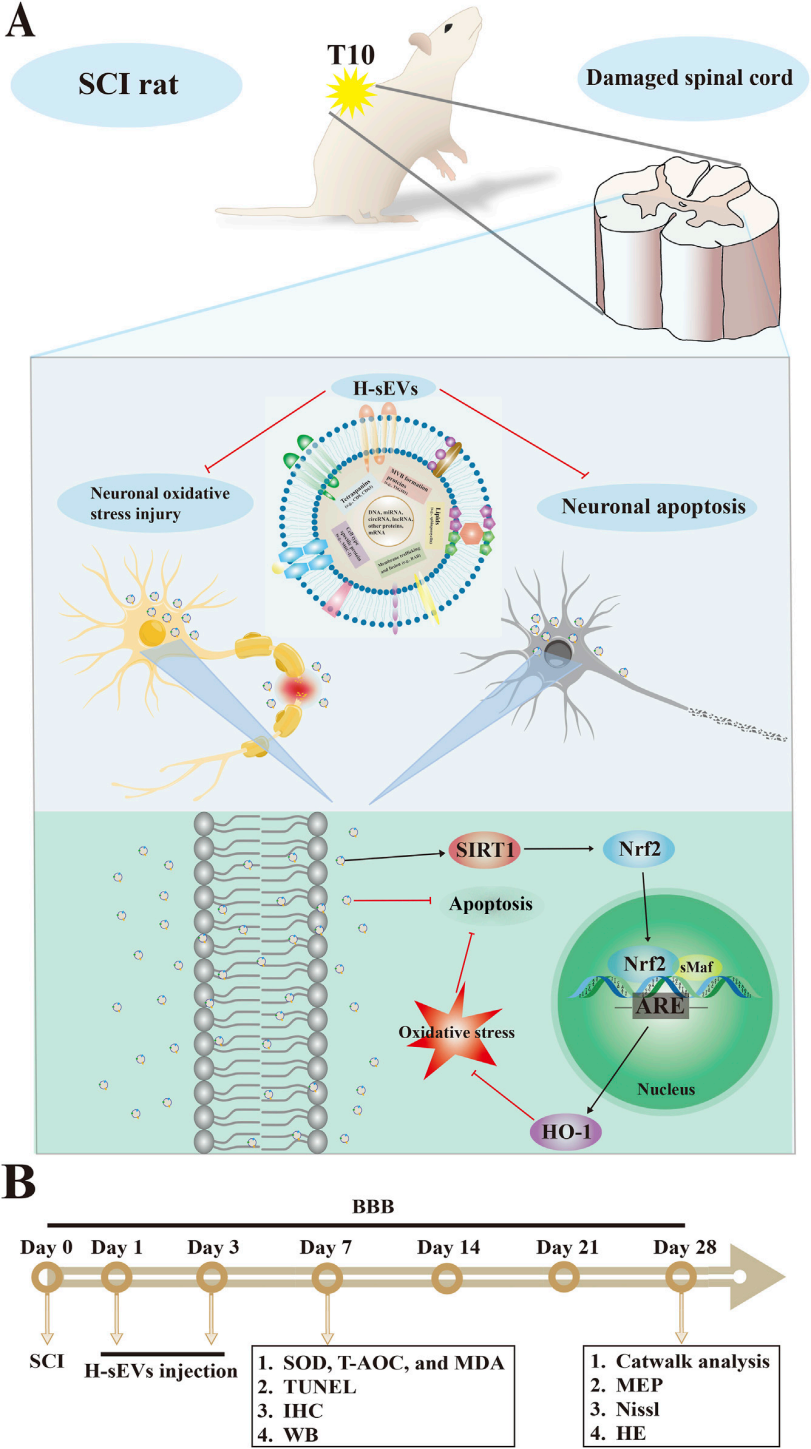
Hypoxic-preconditioned mesenchymal stem cell-derived small extracellular vesicles inhibit neuronal death after spinal cord injury by regulating the SIRT1/Nrf2/HO-1 pathway. **(A)** The mechanism of H-sEVs inhibit neuronal death in SCI repair. **(B)** The scheme outlining the experiments performed.

It has been reported that a variety of cells have been used in clinical treatment of SCI, such as MSCs, neural stem cells (NSCs), Schwann cells, and olfactory ensheathing cells, among which MSCs have the best efficacy (Assinck et al., 2017; Xu et al., 2022; Ribeiro et al., 2023). Because of their biological characteristics and easy to obtain and culture, MSCs have great prospects in regenerative medicine (Si et al., 2011; Tahmasebi and Barati, 2022). However, no matter which cell type is transplanted, the graft will disappear in the host after about 2–3 weeks and will not survive long term (Nakazaki et al., 2021). Therefore, studies have shown that transplanted cells are not the main substances promoting SCI repair, but EVs secreted by cells are the main force (Phan et al., 2018; Nakazaki et al., 2021). At first, EVs were found in blood, but at the time scientists thought “platelet dust” in the blood (Wolf, 1967). With the deepening of research, a large number of studies have proved that EVs have the biological function of targeting receptor cells, such as the delivery of signaling molecules and carrying nanoscale drugs (Thery et al., 2018; Sheng et al., 2021). Currently, researchers like to use stem cells to isolate and purify EVs because of their properties (Kourembanas, 2015). The commonly used methods for EVs extraction include ultracentrifugation, ultrafiltration, and kit extraction (Thery et al., 2018). In our study, we purified EVs by density gradient ultracentrifugation to ensure their integrity and biological function. We then observed the shape and size by TEM, confirmed the concentration and particle size distribution by NTA, and identified the markers CD9, CD63, and TSG101 by WB. The study of Niu et al. (Niu et al., 2022) showed that exosomes derived from bone marrow MSCs could alleviate oxidative stress, inflammation and apoptosis after ischemia reperfusion injury and promote skin flap survival. It has shown that H-sEVs are more consistent with the damage microenvironment than EVs, and that the efficacy after SCI treatment is better (Li et al., 2022). However, the mechanism of its action on SCI oxidative stress response is not clear, and further exploration of its specific targets is still needed to provide theoretical basis for clinical treatment. This is the great significance of this study (Figures 8A, B).

Changes in the microenvironment at the site of injury lead to a series of cascade reactions, and even neurons that survive the primary trauma may be lost in a series of secondary events (such as oxidative stress, apoptosis, and inflammation) (Fan et al., 2018; Fan et al., 2022). When the MDA increases in the microenvironment at the site of injury, the activity of SOD and T-AOC, two endogenous antioxidant enzymes, which are responsible for converting superoxide free radicals into hydrogen peroxide and water, will decrease (Mei et al., 2022). Disorder of oxides or antioxidants in the spinal microenvironment such as MDA, SOD, and T-AOC after injury leads to secondary injury that exacerbates irreparable neurotrauma and permanent loss of function. The changes of microenvironment after SCI are very complex. Therefore, it is very necessary to study the protection of antioxidant stress on the nervous system. In our study, a stable SCI model was established first, followed by early injection of H-sEVs through the caudal vein to treat SCI rats. Fortunately, we found that from day 7 onwards, BBB scores of rats treated with H-sEVs were significantly higher than those in the SCI group. Moreover, by detecting SOD, MDA, and T-AOC in the spinal cord tissue homogenate, we found that SOD and T-AOC significantly increased and MDA significantly decreased after H-sEVs treatment. These results suggest that H-sEVs can relieve early oxidative stress after SCI. It has shown that SIRT1 is an important molecule of antioxidant stress (Mao et al., 2022). Zhong et al. (2021) showed a close correlation between serum SIRT1 content and SCI severity. In this study, it was found that SIRT1 in the spinal cord of SCI rats was significantly increased after H-sEVs treatment, and it was obviously observed that the expression level of SIRT1 in the spinal cord was negatively correlated with the lesion area in the spinal cord, and positively correlated with the recovery of hind limb motor function of rats.

Oxidative stress at the early stage of injury causes neuronal apoptosis, which is one of the key obstacles to SCI recovery (Hall et al., 2016; Coyoy-Salgado et al., 2019). Therefore, reducing the production of oxidative factors after injury and alleviating neuronal apoptosis are considered to be effective methods to promote the recovery of nerve function after SCI. In this study, we found that H-sEVs treatment at the early stage of injury could significantly promote the recovery of motor function and histology in rats after SCI. Our further study found that H-sEVs could significantly reduce the expression of Bax in the injured area of the spinal cord, while the expression of Bcl-2 was significantly upregulated. A large amount of evidence has shown that Bax and Caspase-3 are involved in the pro-apoptotic effect, while Bcl-2 has the anti-apoptotic effect (Raghupathi et al., 2002; Hains et al., 2003). This work provides new evidence that H-sEVs play an important anti-apoptotic role in SCI rats, reducing neuronal apoptosis due to secondary damage. Oxidative stress is thought to be an important factor in cell damage and is usually caused by overproduction of ROS (Marrocco et al., 2017). Under physiological conditions, ROS production level is low and is eliminated by endogenous antioxidant system (Xiong et al., 2023). However, when ROS exceeds the body or cell clearing capacity, oxidative stress damage will occur in the body or cells. Oxidative stress plays an important role in the cascade of pathological changes in SCI. Notably, SIRT1 proteins enhance cellular tolerance to oxidative stress by regulating a variety of genes and their associated signaling pathways. SIRT1 has been shown to regulate inflammation, apoptosis and oxidative stress responses through several signaling pathways to relieve neuronal apoptosis after SCI and protect neural function. In this study, we further verified the anti-oxidative stress and anti-apoptosis effects of H-sEVs on PC12 neuron cells through *in vitro* experiments. We found that H-sEVs could not only reduce the high level of ROS expression in injured PC12 cells, but also inhibit cell apoptosis and improve cell activity. Moreover, we also found that H-sEVs could increase the contents of T-AOC and SOD and decrease the contents of MDA in injured PC12 cells, which indicated that H-sEVs could also play an anti-oxidative stress role in injured PC12 cells.

The results of our *in vitro* study are highly consistent with those observed *in vivo*. However, our *in vivo* studies only found the correlation between SIRT1 and neuronal oxidative stress, and whether it regulates neuronal oxidative stress through SIRT1 remains unknown. Therefore, lentivirus transfection was used to inhibit the expression of SIRT1 in PC12 cells. After H_2_O_2_-induced oxidative stress and H-sEVs treatment, we found that the expression levels of SIRT1 and its downstream signaling molecules Nrf2 and HO-1 in PC12 cells with SIRT1 knockout were significantly reduced compared with normal PC12 cells. In addition, compared with normal cells, the cell activity, SOD, and T-AOC expression levels decreased, while MDA expression levels increased. These results suggest that SIRT1 is a key target for H-sEVs to exert antioxidant stress. H-sEVs may relieve H_2_O_2_-induced oxidative stress and apoptosis in PC12 cells by activating SIRT1/Nrf2/HO-1 signaling pathway protein expression. Among them, SIRT1 can act as the upstream of Nrf2 when regulating the cascade reaction related to oxidative stress, promote the translocation of Nrf2 into the nucleus, increase the activation and transcription of Nrf2, activate the expression of antioxidant enzymes, and enhance the cell response to oxidative stress. Nrf2 is a key component of the antioxidant defense system, which can recognize the promoter sequences of the downstream antioxidant enzymes such as HO-1, SOD, NQO-1, and glutathione peroxidase to regulate oxidative stress caused by reactive oxygen species or nitrogen species. It is not the H-sEVs that activates the signaling pathway, but the miRNA or protein inside the H-sEVs that activates. Our previous study has compared normal EV to hypoxic EV (Yang et al., 2023), and found that the efficacy of H-sEVs was superior. However, the specific mechanism of action remains unclear. This study demonstrated *in vivo* and *in vitro* that H-sEVs can inhibit oxidative stress and apoptosis of neurons by up-regulating SIRT1 expression. Our results provide new intervention targets and ideas for H-sEVs to relieve early apoptosis and oxidative stress after SCI.

However, we acknowledge some limitations of our study. Firstly, the expression level of SIRT1 in neurons or glial cells in spinal cord tissue was not determined, as it is expressed in all cell types. The focus of this study is on the role of SIRT1 protein in SCI, rather than its relationship with various cells. Secondly, PC12 cells were used instead of primary neurons *in vitro*. However, primary neurons should be utilized when studying specific signaling pathways. Finally, this study only employed lentivirus transfection to inhibit SIRT1 expression in PC12 cells. Further verification of its role could be achieved by knocking out SIRT1 *in vivo*. Additionally, the integrity of this pathway can be confirmed through both *in vivo* and *in vitro* rescue experiments involving downstream proteins Nrf2 and HO-1 regulated by this pathway.

## Conclusion

In conclusion, we demonstrated that H-sEVs can alleviate oxidative stress and apoptosis in the microenvironment after SCI, and elucidated the underlying molecular mechanisms. More importantly, the important role of the key protein SIRT1 in H-sEVs treatment of SCI was demonstrated through *in vivo* and *in vitro* experiments. This study provides a promising therapeutic means for the clinical treatment of SCI and lays a foundation for the clinical application of H-sEVs.

## Data Availability

The original contributions presented in the study are included in the article/supplementary material, further inquiries can be directed to the corresponding authors.
