# Supplementary material for: Hypoxic-preconditioned mesenchymal stem cell-derived small extracellular vesicles inhibit neuronal death after spinal cord injury by regulating the SIRT1/Nrf2/HO-1 pathway
Source: Front Pharmacol. 2024 Aug 23;15:1419390. doi: 10.3389/fphar.2024.1419390 (PMC11377843; doi:10.3389/fphar.2024.1419390)
Supplement: Supplementary file 1 [file DataSheet1.PDF]

Table 1. Reagents

Antibody	Company	Cat#	Concentration
Primary antibodies			
CD29	ThermoFisher Scientific	12-0291-82	1:200
CD90	ThermoFisher Scientific	17-0900-82	1:200
CD34	ThermoFisher Scientific	11-0341-82	1:200
CD45	BD Bioscience	561587	1:200
CD9	Merck	SAB4503606	1:1000
CD63	ThermoFisher Scientific	PA5-92370	1:1000
TSG101	ThermoFisher Scientific	MA1-23296	1:1000
Bcl-2	Abcam	ab196495	1:1000
Bax	Abcam	ab32503	1:2000
β-actin	ThermoFisher Scientific	MA1-140	1:10000
SIRT1	Santa Cruz Biotechnology	sc-74465	IHC (1:200)<br>WB (1:1000)
Nrf2	ThermoFisher Scientific	PA5-27882	1:1000
HO-1	Abcam	ab68477	1:10000
Secondary antibodies			
Goat anti-Mouse IgG (H+L), HRP	ThermoFisher Scientific	31430	1:10000
Goat anti-Rabbit IgG (H+L), HRP	ThermoFisher Scientific	31460	1:10000

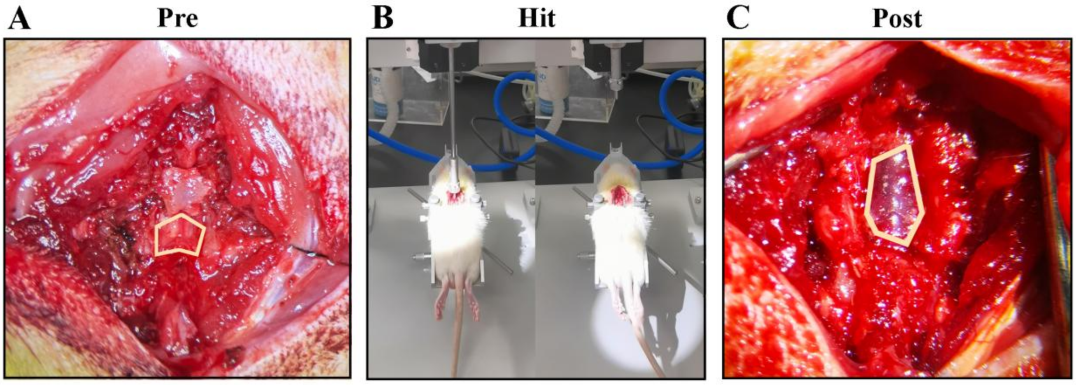

Figure S1. Established SCI model in SD rat. (A) Pre-hit, T10 spinal cord exposed under microscope (yellow box). (B) Spasticity of lower limbs and tail rotation after impact. (C) Post-hit, a significant hematoma observed microscopically at the T10 (yellow box).

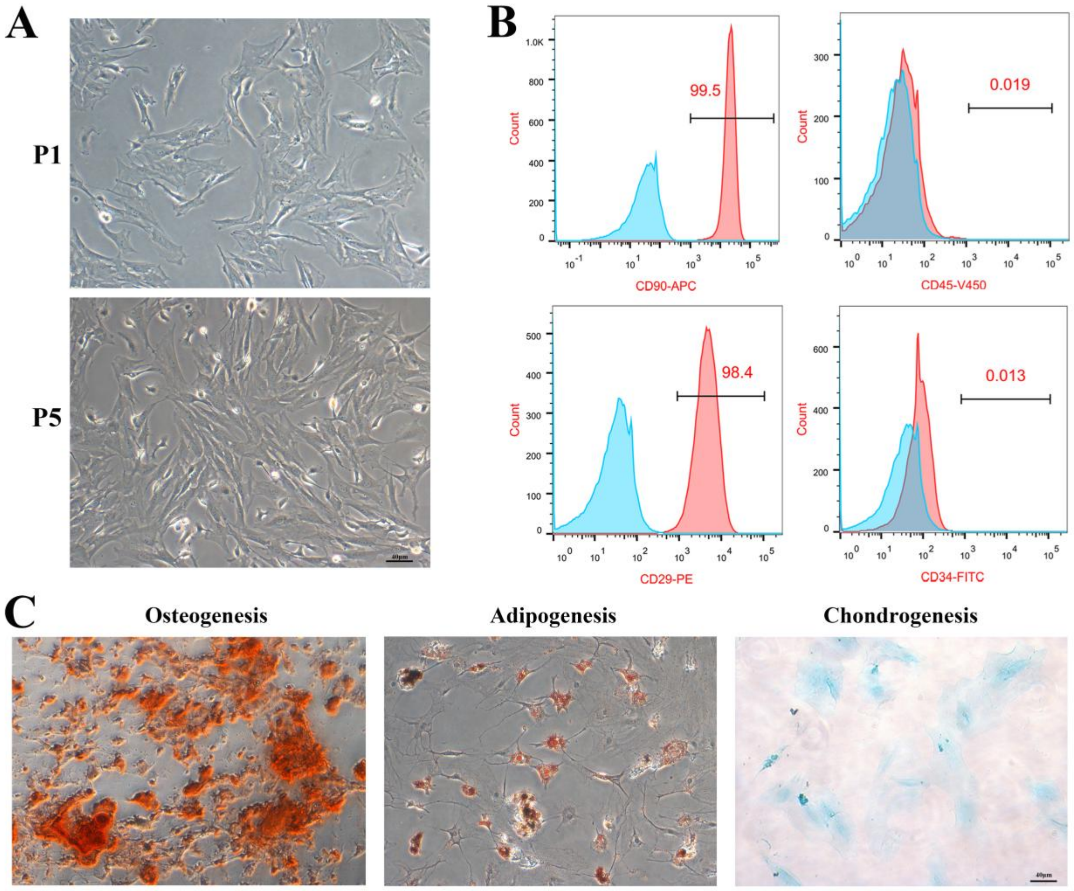

Figure S2. Identification of the purity and differentiation capacity of MSCs. (A) Representative images of P1 and P5 MSCs in light microscopy (20×). (B) MSCs appraisal of flow cytometry. (C) Representative image about differentiation identification of MSCs in light microscope (20×). (Scale bar: 40 μm)

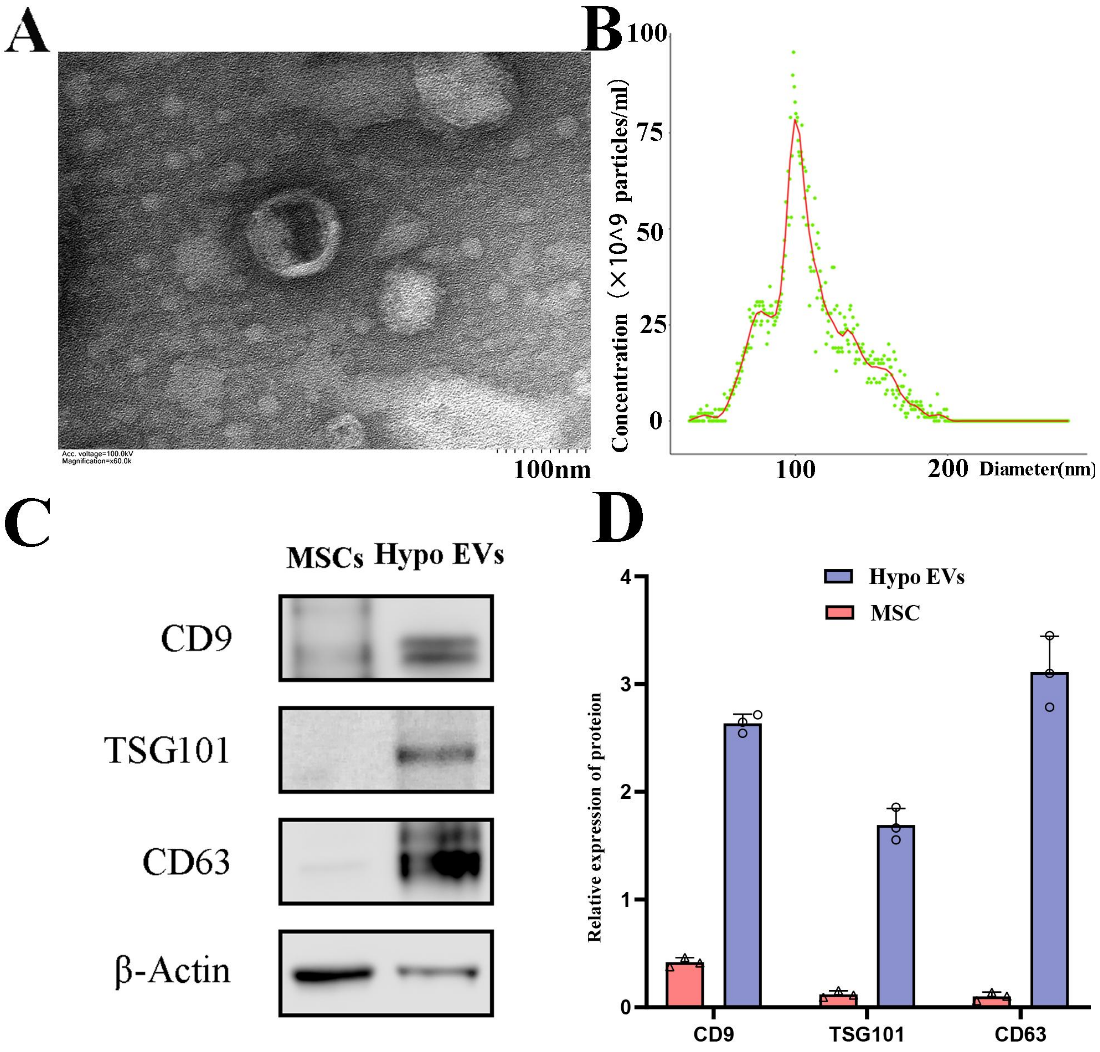

Figure S3. Characterization of H-sEVs. (A) Morphology of H-sEVs shown by TEM (Scale bar: 100nm). (B) Particle size distribution measured by NTA. (C) Representative WB image of H-sEVs positive markers (CD9, CD63, and TSG101; internal reference protein: β-actin). (D) Statistical analysis of relative expression of H-sEVs positive markers (CD9, CD63, and TSG101). (n = 3, *$p < 0.05$, **$p < 0.01$, ***$p < 0.001$, ns: not significant)

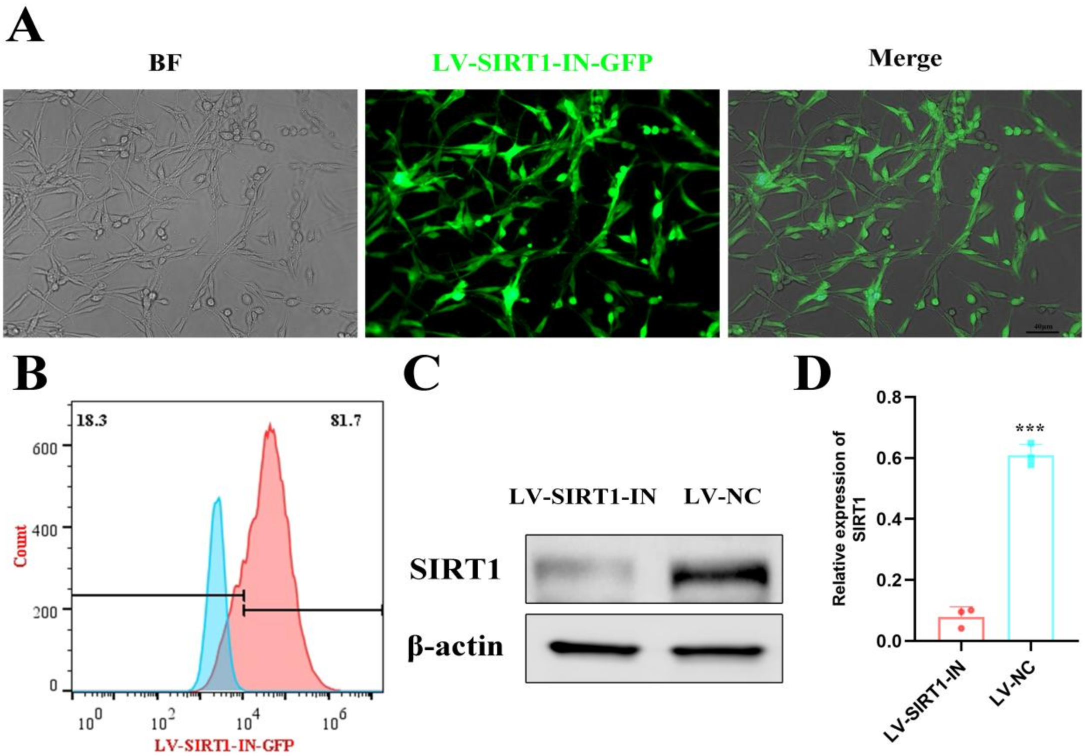

Figure S4. Knockdown efficiency of SIRT1 in PC12 cells. (A) Representative image of SIRT1 inhibition in PC12 cells transfected with LV-SIRT1-IN-GFP (Scale bar: 40 μm). (B) Flow analysis of SIRT1 inhibition in PC12 cells transfected with LV-SIRT1-IN-GFP. (C) Representative WB image of SIRT1 knockdown in PC12 cells (internal reference protein: β-actin). (D) Statistical analysis of relative expression of SIRT1 in PC12 cells. (n = 3, *$p < 0.05$, **$p < 0.01$, ***$p < 0.001$, ns: not significant)
